# Supplementary material for: Discrete choice experiment exploring women’s preferences in a novel device designed to monitor the womb environment and improve our understanding of reproductive disorders
Source: BMJ Open. 2022 Apr 29;12(4):e058419. doi: 10.1136/bmjopen-2021-058419 (PMC9058797; doi:10.1136/bmjopen-2021-058419)
Supplement: Supplementary data [file bmjopen-2021-058419supp001.pdf]

Investigations for women with reproductive problems.

A questionnaire study.

What are patients' preferences for a novel device able to monitor the womb environment?

Introduction

Doctors and researchers at the University of Southampton in collaboration with VivoPlex Medical Ltd are developing and carrying out research into a novel monitoring device that may be offered to women with fertility issues. Whilst this device has not yet been produced, we are interested in your views about this new sensor device, and what features would be important to you. Participating in this would involve you completing the attached questionnaire. Once this questionnaire is complete and handed to a member of staff, you are not required to do anything further. Responses will be anonymous and confidential. Your views would help us to develop and tailor this novel sensor for women planning a pregnancy in the future. It is important to understand this device is not yet available to you.

We would like you to *imagine* that your doctor has given you the option of using a sensor device in order to find out more about your womb environment. Once the sensor device is inserted into the womb, you would also be asked to wear a garment close to your womb, for a number of days, that holds a small battery pack and information receiver, all supplied to you along with the sensor. The sensor works by measuring three characteristics of your womb environment continuously, and sending that information back to the receiver. Your doctor would discuss the information with you which may allow them to guide you in making decisions about your next treatment steps towards a successful pregnancy.

Ng KYB, *et al. BMJ Open* 2022; 12:e058419. doi: 10.1136/bmjopen-2021-058419

Patient information about the study

What is the purpose of the study?

This is a questionnaire study to help us understand what women consider the most important features of the device. By completing the following questionnaire you could help us to develop important features of this novel test.

Why have I been chosen and do I need to take part?

As you are attending the outpatient clinic and hoping to have a baby in the future, we would be interested in your opinions about this novel device. We are asking if you would be prepared to complete this questionnaire. You can decide whether or not to take part, and your decision will have no impact on your care.

What will happen to me if I decide to take part?

Completing the attached questionnaire will take around 10 minutes, and once completed you should return it to the 'questionnaire collection box' at reception. There will be no follow up after the questionnaire has been handed in, and your answers will be confidential and anonymous, and accessed only by members of the research team. The questionnaire asks questions around sensitive topics including fertility and conception and if you do not feel comfortable answering them, you can withdraw from the study at any time without your clinical care being affected.

What will happen to the results of the research?

The findings will be presented at scientific meetings, nationally and internationally, published in medical journals, and possibly in the local and national press. You will not be identified in these reports or publications. The University policy for data protection (https://www.southampton.ac.uk/about/governance/policies/privacy-policy.page) will be followed.

Who is organising and funding the research?

This study is funded by the National Institute for Health Research (NIHR) Invention for Innovation stream which supports clinical development of medical devices in areas of existing or emerging patient need. The study is being led by Professor Ying Cheong from the University of Southampton. This study has been reviewed and approved by X Ethics Committee (Ethics No.: to be confirmed).

Contact for further information.

For further information please contact Dr Bonnie Ng at the University of Southampton, email: bonnie.ng@soton.ac.uk. Or ask to speak to a member of the reproductive health research team 023 8120 6856

Information on your data

The University of Southampton is the sponsor or this study based in the United Kingdom, Southampton. We will be using information from you in order to undertake this study and will act as the data controller for this study. This means that we are responsible for looking after your information and using it properly. The University of Southampton will keep identifiable information about you for 10 years after the study has finished.

Your rights to access, change or move your information are limited, as we need to manage your information in specific ways in order for the research to be reliable and accurate. If you withdraw from the study, we will keep the information about you that we have already obtained. To safeguard your rights we will use the minimum personally identifiable information possible.

You can find more about how we use your information at:

http://www.calendar.soton.ac.uk/sectionIV/research-data-management.html

The University of Southampton will collect information from you for this research study in accordance with our instructions.

The University Hospitals Southampton NHS Foundation Trust will keep your name, and contact details confidential and will not pass this information to the University of Southampton. The University Hospitals Southampton NHS Foundation Trust will use this information as needed, to contact you about the research study, and make sure that relevant information about the study is recorded for your care, and to oversee the quality of the study. Certain individuals from the University of Southampton and regulatory organisations may look at your medical and research records to check the accuracy of the research study. The University of Southampton will only receive information without any identifying information The people who analyse the information will not be able to identify you and will not be able to find your name or contact details.

The University Hospitals Southampton NHS Foundation Trust will keep identifiable information about you from this study for 10 years after the study has finished.

Please pull off the front 2 pages and keep for future reference

UNIVERSITY OF Southampton VivoPlex University Hospital Southampton NHS Foundation Trust NHS National Institute for Health Research

CONSENTING TO THE QUESTIONNAIRE.

If you have any further questions please ask a member of staff to contact the research team on 02381206856.

By ticking this box ☐ you confirm that:

- You have read the information at the start of this questionnaire.
- You understand who to contact with any questions.
- You understand participation in completely voluntary.
- You are over 18 and under 50 years old.
- You are attending an outpatients appointment at the Princess Anne Hospital.

Study ID ☐

When you have completed this questionnaire, please return to the 'Questionnaire collection box' at reception.

If you have partially completed the questionnaire or prefer not to take part, you can also return your questionnaire in the same box.

Thank you for your time

EXPLANATION ABOUT THE QUESTIONNAIRE.

In this questionnaire you will be presented with several **hypothetical** scenarios in which two sensors with different characteristics will be described. In each situation, we would like you to choose which sensor you prefer out of two options: Device **A** or Device **B**.

Each sensor will be described to you in terms of four features:

1. **Length of time using the device.** The number of days the device would remain inside your womb. Longer time in your womb could give more information to the doctor. There will be three choices, ***7 days, 14 days, or 28 days***

2. **Information obtained from the device and its use in guiding treatment.** There are three choices, the information obtained will guide treatment in a ***limited*** number of cases, ***majority*** of cases or ***all*** cases.

3. **Risk of complications.** There may either be short- or long-term complications from using the device. The long-term effects of using the device are currently unknown, and further clinical trials will be conducted to explore this. The short-term complications could be an infection needing antibiotic treatment, pain or discomfort needing pain relief or expulsion (device falling out of the womb). There will be two choices, 1 in 100 women (***1%, lower risk***), or 10 in 100 women (***10%, higher risk***).

4. **Discreteness of the information receiver.** Once the sensor is inserted into the womb, you will be asked to wear a garment which holds the information receiver. This receiver is required for the data on the womb environment to be obtained and interpreted by a clinician. There are three options regarding the discreteness if the information receiver that you will be asked to wear- ***completely discrete*** (i.e. even when tight clothing is worn the receiver will not be noticeable to others), ***moderately discrete*** (i.e. the receiver will be noticeable to others when tight clothing is worn), and ***indiscrete*** (i.e. the receiver will be noticeable to others irrespective of the type of clothing worn).

UNIVERSITY OF Southampton   VivoPlex   NHS University Hospital Southampton NHS Foundation Trust   NHS National Institute for Health Research

HOW TO COMPLETE THIS QUESTIONNAIRE.

There are no right or wrong answers - we are just interested in your views. Please choose the option that in your opinion is the better device, the one that best matched your priorities.

EXAMPLE QUESTION

We will now take you through an example question. You will not need to answer the question in this section.

Consider the devices described by the following two sets of features (Device **A** and Device **B**). Please indicate which choice you would prefer by circling the column.

	**Device A**	**Device B**
**Length of time using the device**	7 Days	28 Days
**Information obtained from the device and its use in guiding treatment**	The information obtained will guide treatment in all cases	The information obtained will guide treatment in all cases
**Risk of complications**	10%	1%
**Discreteness of the information receiver**	Moderately discrete	Completely discrete

In this example, Device A has been selected. The person made the choice based on the features of Device A and Device B, and decided her preferred choice.

CHOICE SETS

Ng KYB, *et al. BMJ Open* 2022; 12:e058419. doi: 10.1136/bmjopen-2021-058419

Please answer **ALL** 9 choice sets and indicate your choice by drawing a circle around your preferred device.

Choice Set 1		
	**Device A**	**Device B**
**Length of using the device**	7 Days	28 Days
**Information obtained from the device and its use in guiding treatment**	The information obtained will guide treatment in a limited number of cases	The information obtained will guide treatment in a limited number of cases
**Risk of complications**	1%	10%
**Discreteness of the information receiver**	Completely discrete	Indiscrete

Choice Set 2		
	**Device A**	**Device B**
**Length of using the device**	28 Days	7 Days
**Information obtained from the device and its use in guiding treatment**	The information obtained will guide treatment in the majority of cases	The information obtained will guide treatment in the majority of cases
**Risk of complications**	10%	1%
**Discreteness of the information receiver**	Completely discrete	Moderately discrete

Choice Set 3		
	**Device A**	**Device B**
**Length of using the device**	7 Days	14 Days
**Information obtained from the device and its use in guiding treatment**	The information obtained will guide treatment in a limited number of cases	The information obtained will guide treatment in a limited number of cases
**Risk of complications**	1%	10%
**Discreteness of the information receiver**	Indiscrete	Completely discrete

Choice Set 4		
	**Device A**	**Device B**

Ng KYB, et al. BMJ Open 2022; 12:e058419. doi: 10.1136/bmjopen-2021-058419

University of Southampton | VivoPlex | NHS University Hospital Southampton NHS Foundation Trust | NHS National Institute for Health Research

**Length of using the device**	28 Days	14 Days
**Information obtained from the device and its use in guiding treatment**	The information obtained will guide treatment in a limited number of cases	The information obtained will guide treatment in all cases
**Risk of complications**	10%	10%
**Discreteness of the information receiver**	Moderately discrete	Completely discrete

Choice Set 5		
	**Device A**	**Device B**
**Length of using the device**	14 Days	28 Days
**Information obtained from the device and its use in guiding treatment**	The information obtained will guide treatment in all cases	The information obtained will guide treatment in all cases
**Risk of complications**	1%	10%
**Discreteness of the information receiver**	Moderately discrete	Completely discrete

Choice Set 6		
	**Device A**	**Device B**
**Length of using the device**	7 Days	28 Days
**Information obtained from the device and its use in guiding treatment**	The information obtained will guide treatment in all cases	The information obtained will guide treatment in all cases
**Risk of complications**	10%	1%
**Discreteness of the information receiver**	Moderately discrete	Indiscrete

Ng KYB, *et al. BMJ Open* 2022; 12:e058419. doi: 10.1136/bmjopen-2021-058419

Choice Set 7		
	**Device A**	**Device B**
**Length of using the device**	14 Days	28 Days
**Information obtained from the device and its use in guiding treatment**	The information obtained will guide treatment in the majority of cases	The information obtained will guide treatment in the majority of cases
**Risk of complications**	10%	1%
**Discreteness of the information receiver**	Indiscrete	Moderately discrete

Choice Set 8		
	**Device A**	**Device B**
**Length of using the device**	14 Days	7 Days
**Information obtained from the device and its use in guiding treatment**	The information obtained will guide treatment in a limited number of cases	The information obtained will guide treatment in a limited number of cases
**Risk of complications**	1%	10%
**Discreteness of the information receiver**	Moderately discrete	Completely discrete

Choice Set 9		
	**Device A**	**Device B**
**Length of using the device**	14 Days	14 Days
**Information obtained from the device and its use in guiding treatment**	The information obtained will guide treatment in a limited number of cases	The information obtained will guide treatment in all cases
**Risk of complications**	1%	1%
**Discreteness of the information receiver**	Indiscrete	Completely discrete

Ng KYB, *et al. BMJ Open* 2022; 12:e058419. doi: 10.1136/bmjopen-2021-058419

Section 4: ABOUT YOURSELF

Age ☐ **First 4 characters of your postcode** ☐☐☐☐

Relationship status

Single ☐
In a relationship and living apart ☐
In a relationship and living together ☐
Engaged ☐
Married ☐
Civil partnership ☐
Other ☐
Please state_________________________

Education attainment

Higher education & professional/vocational equivalents ☐
A levels, vocational level 3 and equivalents ☐
GCSE/O level grade A*-C, vocational level 2 and equivalents ☐
Qualifications at level 1 and below ☐
Other qualifications: level unknown (including foreign qualifications) ☐
No qualifications ☐

What is your ethnic group?

White ☐
Mixed/multiple ethnic groups ☐
Asian/Asian British ☐
Black/African/Caribbean/Black British ☐
Other Ethnic group ☐
Please state______________________________

Are you needing treatment for infertility/subfertility?.

Yes ☐ No ☐

Are you needing treatment for recurrent miscarriages (3 or more consecutive early miscarriages)?

Yes ☐ No ☐

Do you or your partner have children?

Yes ☐ No ☐

Ng KYB, *et al. BMJ Open* 2022; 12:e058419. doi: 10.1136/bmjopen-2021-058419

Have you had any of the following treatment?

Hysteroscopy (procedure to look inside the womb) ☐

Diagnostic laparoscopy (keyhole procedure to look inside the abdomen only) ☐

Operative laparoscopy (keyhole procedure to look inside the abdomen AND treat pathology) ☐

NHS funded IVF ☐

Privately funded IVF ☐

Do you qualify for NHS funded IVF?

Yes ☐ No ☐ Don't know ☐

Section 5: FEEDBACK (optional)

How easy or difficult did you find it to answer these questions? Please put an 'X' by your response:

Very easy	
Quite easy	
Neither easy nor difficult	
Quite difficult	
Very difficult	

Please write any comments you would like to make about this questionnaire:

____

____

____

____

____

Do you have any ideas on how to improve the questionnaire or the product?

____

____

____

____

____

Thank you for taking the time to complete this questionnaire. Your answers will help us determine how to better develop a novel sensor device designed for women planning a pregnancy.

Ng KYB, *et al. BMJ Open* 2022; 12:e058419. doi: 10.1136/bmjopen-2021-058419
