# Supplementary material for: Discrete choice experiment exploring women’s preferences in a novel device designed to monitor the womb environment and improve our understanding of reproductive disorders
Source: BMJ Open. 2022 Apr 29;12(4):e058419. doi: 10.1136/bmjopen-2021-058419 (PMC9058797; doi:10.1136/bmjopen-2021-058419)
Supplement: Author's manuscript [file bmjopen-2021-058419.draft_revisions.pdf]

BMJ Open

BMJ Open is committed to open peer review. As part of this commitment we make the peer review history of every article we publish publicly available.

When an article is published we post the peer reviewers' comments and the authors' responses online. We also post the versions of the paper that were used during peer review. These are the versions that the peer review comments apply to.

The versions of the paper that follow are the versions that were submitted during the peer review process. They are not the versions of record or the final published versions. They should not be cited or distributed as the published version of this manuscript.

BMJ Open is an open access journal and the full, final, typeset and author-corrected version of record of the manuscript is available on our site with no access controls, subscription charges or pay-per-view fees (http://bmjopen.bmj.com).

If you have any questions on BMJ Open's open peer review process please email info.bmjopen@bmj.com

BMJ Open

# Women's preferences in a novel intrauterine device designed to monitor the womb environment in real time: A discrete choice experiment

| | |
|---|---|
| Journal: | *BMJ Open* |
| Manuscript ID | bmjopen-2021-058419 |
| Article Type: | Original research |
| Date Submitted by the Author: | 18-Oct-2021 |
| Complete List of Authors: | Ng, Ka Ying Bonnie; University of Southampton Faculty of Medicine, School of Human Development and Health; Princess Anne Hospital, Department of Obstetrics and Gynaecology<br>Evans, Rhiannon; Salisbury NHS Foundation Trust, Obstetrics and Gynaecology Department<br>Mentzakis, Emmanouil; University of Southampton, Department of Economics<br>Cheong, Ying; University of Southampton Faculty of Medicine, School of Human Development and Health; Princess Anne Hospital, Department of Obstetrics and Gynaecology |
| Keywords: | GYNAECOLOGY, HEALTH ECONOMICS, REPRODUCTIVE MEDICINE, Subfertility < GYNAECOLOGY |
| | |

SCHOLARONE™
Manuscripts

*I, the Submitting Author has the right to grant and does grant on behalf of all authors of the Work (as defined in the below author licence), an exclusive licence and/or a non-exclusive licence for contributions from authors who are: i) UK Crown employees; ii) where BMJ has agreed a CC-BY licence shall apply, and/or iii) in accordance with the terms applicable for US Federal Government officers or employees acting as part of their official duties; on a worldwide, perpetual, irrevocable, royalty-free basis to BMJ Publishing Group Ltd ("BMJ") its licensees and where the relevant Journal is co-owned by BMJ to the co-owners of the Journal, to publish the Work in this journal and any other BMJ products and to exploit all rights, as set out in our licence.*

*The Submitting Author accepts and understands that any supply made under these terms is made by BMJ to the Submitting Author unless you are acting as an employee on behalf of your employer or a postgraduate student of an affiliated institution which is paying any applicable article publishing charge ("APC") for Open Access articles. Where the Submitting Author wishes to make the Work available on an Open Access basis (and intends to pay the relevant APC), the terms of reuse of such Open Access shall be governed by a Creative Commons licence – details of these licences and which Creative Commons licence will apply to this Work are set out in our licence referred to above.*

*Other than as permitted in any relevant BMJ Author's Self Archiving Policies, I confirm this Work has not been accepted for publication elsewhere, is not being considered for publication elsewhere and does not duplicate material already published. I confirm all authors consent to publication of this Work and authorise the granting of this licence.*

For peer review only

**Women's preferences in a novel intrauterine device designed to monitor the womb environment in real time: A discrete choice experiment**

Ka Ying Bonnie Ng[1,2*] Rhiannon Evans[3] Emmanouil Mentzakis[4] Ying Cheong[1,2]

[1]Faculty of Medicine, University of Southampton, SO17 1BJ

[2]Obstetrics and Gynaecology department, Princess Anne Hospital, Southampton University Hospitals NHS Foundation Trust, Coxford Road, Southampton SO16 5YA

[3]Obstetrics and Gynaecology Department, Salisbury District Hospital, Salisbury NHS Foundation Trust, Odstock Road, Salisbury, SP2 8BJ

[4]Department of Economics, University of Southampton, SO17 1BJ

*Corresponding author

bonnie.ng@doctors.org.uk (BN)

For peer review only

**Abstract**

Objectives

To determine the relative importance of key attributes of a novel intrauterine monitoring device in women with reproductive disorders.

Design

A Discrete Choice Experiment (DCE) was used as a quantitative measure for eliciting individual preferences in this novel investigative tool. The attributes used in the choice scenarios were length of time using the device, information obtained to guide treatment, risk of complications and discreteness.

Setting

Secondary care hospital in Hampshire, UK

Participants

361 women of reproductive age (18-50 years), attending the gynaecology outpatient department wishing to achieve a pregnancy

Primary and secondary outcome measures

Analysis using conditional logit and latent class logit regression models to determine the preference for each attribute.

Results

Conditional logit co-efficients allow comparison between attributes with women placing the most importance on obtaining information to guide treatment in all cases (2.771), followed by


For peer review only

having a completely discrete device (1.104), reducing risk of complications by 1% (0.184) and lastly decreased length of time by 1 day (0.0150). All co-efficients $p<0.01$.

Latent class conditional logit assigns participants to two classes with 27.4% in Class 1 who are less likely to have higher education (co-efficient -0.660, 95% CI -0.967 and -0.353), and less likely to qualify for NHS funded IVF (co-efficient -0.709, 95% CI -1.065 and -0.353) compared to Class 2. Those in Class 2 placed 1.7 times more importance on a device whose information guided treatment in all cases and a 1% decrease in complications risk was nearly 15 times more attractive.

Conclusions

Women placed most importance on having a device that obtains information to guide treatment and are willing to use the device for a longer length of time, have a device with higher risk of complications and an indiscrete device if it is able to provide answers and direction for treatment of their reproductive disorder.

For peer review only

**Strengths and Limitations of the Study**

- This discrete choice experiment is the first to elicit patient preferences for the attributes/characteristics of a novel intrauterine monitoring device as an investigative tool in women with reproductive failures, .
- The attributes and levels for the novel medical device were determined following focus groups and discussions with potential users, experts, clinicians and researchers.
- Participants were recruited in a single UK hospital, which may limit the applicability in other parts of the UK or in other countries.
- Inherent to DCE, participants do not experience the resulting consequences of their decisions, therefore their responses may not accurately reflect real choices.
- We have not included a cost attribute as our aim was to explore the trade-offs between the attributes which seemed most important to users rather than economic analysis.

For peer review only

## Introduction

The development and introduction of novel technologies, diagnostic tools and therapies in the area of clinical medicine is rapidly increasing. Within reproductive health, we have seen the recent introduction of technological tools such as Ovusense®, skin or vaginal sensors designed to monitor menstrual cycles and track ovulation whilst a woman is trying to conceive and AneVivo, a novel device that enables in vivo fertilisation to take place in the maternal womb instead of a laboratory incubator for women who are having assisted reproductive technologies (ART). Despite the rapid development of novel diagnostic and therapeutic tools, including medical devices to assist with conception and pregnancy, there is limited data exploring user's preferences. Engagement of users in the development of novel devices is lacking yet hugely important to inform scientists, researchers, clinicians and policy makers.

Within health economics, Discrete Choice Experiment (DCE) is a quantitative technique used for eliciting individual preferences allowing researchers to explore how individual's value attributes of a programme, product or service. DCEs proceed by asking individuals to state their choice over different hypothetical alternatives over a series of scenarios. It is used increasingly in health services research, primarily to assess patient-stated preferences and willingness to pay for different models of health service delivery (1-3). Within the area of reproductive medicine, DCEs have been used to elicit the preferences placed on fertility treatment (4), including the value of patient centred care (5, 6) and willingness to accept risk and complications of fertility treatment (7).

Recently, an implantable batteryless sensor, which can be placed within the uterus to measure oxygen, pH and temperature has been developed in collaboration with a technology company (Vivoplex, Patent number PCT/GB2017/050609) and University of Southampton (UoS) (Figure 1).

For peer review only

| Attributes | Description | Levels |
|---|---|---|
| **Length of time using the device** | The number of days the device would remain inside your womb. Longer time in your womb could give more information to the doctor. There are 3 choices. | 7 days |
| | | 14 days |
| | | 28 days |
| **Information obtained from the device and its use in guiding treatment** | There are three choices, the information obtained will guide treatment in a ***limited*** number of cases, ***majority*** of cases or ***all*** cases. | Limited number of cases |
| | | Majority of cases |
| | | All cases |
| **Risk of complications** | There may either be short- or long-term complications from using the device. The long-term effects of using the device are currently unknown, and further clinical trials will be conducted to explore this. The short-term complications could be an infection needing antibiotic treatment, pain or discomfort needing pain relief or expulsion (device falling out of the womb). There will be two choices. | 1% Lower risk |
| | | 10% Higher risk |
| **Discreteness of the information receiver** | Once the sensor is inserted into the womb, you will be asked to wear a garment which holds the information receiver. There are three options | completely discrete= even when tight clothing is worn the receiver will not be noticeable to others |
| | | moderately discrete= the receiver will be noticeable to others when tight clothing is worn |
| | | completely discrete= even when tight clothing is worn the receiver will not be noticeable to others |

**Table 1** Attributes and levels for the novel intrauterine monitoring device.

For peer review only

The device is similar in shape and size to a contraceptive coil and has an accompanying wearable garment with an information receiver to collect data. This device has the potential to help personalise and streamline care for women with reproductive disorders such as infertility or repeated miscarriages. A number of key attributes including availability of the device, safety, effectiveness as an investigative tool and inconvenience were previously identified from early Patient and Public Involvement (PPI) work. However, there remains a need to understand what value potential users place on such key attributes, and to explore the relative importance of each attribute if this novel intrauterine device is to be offered as an investigative tool for women affected by reproductive failures in the future.

The aim of this study is to elicit patient preferences for the attributes/characteristics of a novel intrauterine monitoring device as an investigative tool in women with reproductive failures by using a DCE. Insight into the value that women place on key attributes can assist with the further design or development of the device, and the design of clinical trials using the device. The findings may also guide researchers and clinicians designing novel devices within the area of reproductive medicine.

For peer review only

## Methods

### Study Design

This study used a Discrete Choice Experiment (DCE) to elicit patient preferences regarding a novel intrauterine investigative tool to help them with the treatment of their infertility/recurrent miscarriages.

### Development of attributes and levels

Each hypothetical sensor was be described to the participant in terms of four attributes ((1) length of time using the device, (2) information obtained from the device and its use in guiding treatment, (3) risk of complications and (4) discreteness of the information receiver) summarised in Table 1. These were derived from a Public and Patient Involvement (PPI) session where women with reproductive failures (subfertility or repeated miscarriages) were invited to a focus group session. Within the focus groups, their views of the device and their potential involvement in a clinical trial using the device was explored. Along with the PPI session, a literature search and discussion with experts (3 clinicians and researchers working within reproductive medicine and 1 health economist) confirmed the attributes which were of importance. The attributes and DCE questionnaire were further refined through preliminary testing of the questionnaire with potential users of the device.

### Discrete choice experiment design

The combination of attributes and levels resulted in 54 (3x3x2x3) possible scenarios. However, given cognitive burden and fatigue for the respondents, the number of questions to be asked had to be reduced and hence we opted for a fractional factorial design. For this study we were interested in estimating effects for four attributes as well as all two-way

For peer review only

interactions with the information attribute. As such the number of estimable parameters to be identified is 18 (i.e. 17 plus 1 for the constant). A D-efficient fractional factorial design with 18 pair-wise choice-sets was created in SAS software. Literature suggests that 18 choices per individual is possibly more than respondents can handle, potentially impacting the quality of responses (5). To reduce cognitive burden, the design was blocked into two blocks of 9 choice-sets each. At the end of the experiment a series of questions on patients' demographic and medical characteristics was included.

The preliminary DCE questionnaire was piloted on 10 women within the Obstetrics and Gynaecology Department. The women were interviewed; the questionnaire's comprehensibility, usability, amount of choice sets and content validity of the attributes and levels were discussed. After the pilot testing, changes were implemented and repeat testing was carried out. Feedback from the pilot testing shaped the exact formulation of the attributes and the DCE instructions on final DCE questionnaire (Appendix 1).

**Recruitment of participants**

Participants were women of reproductive age (18 to 50 years), who were wishing to achieve a pregnancy now or sometime in the future. Exclusion criteria included those with limited English or limited ability to understand the study design and questionnaire. Participants who met the inclusion and exclusion criteria were recruited from gynaecology clinics within Princess Anne Hospital, Southampton. Women who agree to participate in the study were asked to complete the DCE questionnaire.

Precise power calculation for DCEs have not been developed but some rule-of-thumb approaches are available (8). The first suggest that there are at least 50 respondents per alternative, i.e. 100 for our design. The second, posits that sample size should be larger than 500*c/(t * a), where c is the largest number of levels of attribute (or levels of interaction), t is

For peer review only

the number of choice tasks and a is the number of alternatives. Hence, for main effects only estimation we would need around 42 individuals per block (i.e. 84 in total), while for main plus our specified two-way interactions we would need around 125 individuals per block (i.e. 250 in total).

**Data collection**

The study was approved by HRA and Health and Care Research Wales (HCRW) (Research Ethics Committee reference 19/IE08/0036). The choice sets were administered in an anonymous hard copy of the questionnaire (Appendix 1), which began with participant information for the study and the intrauterine monitoring device. The next section asked for the participant to consent to the questionnaire. Each participant was assigned a study ID. The following sections included explanation about the questionnaire (including the attributes and levels), how to complete the questionnaire, an example question and then 9 choice sets. Each choice set consisted of two novel intrauterine device alternatives described according to the four attributes in the format as in table 2.

| | **Device A** | **Device B** |
|---|---|---|
| **Length of time using the device** | **7** Days | **28** Days |
| **Information guides treatment** | **All** women | **All** women |
| **Risk of complications** | **10%** | **1%** |
| **Discreteness** | **Indiscrete** | **Moderately discrete** |

**Table 2** An example choice set presented to participants. Within each choice set, participants are asked to consider the devices described by the two sets of attributes and choose between device A or device B. Each hypothetical device presented will vary in the four attirbutes: length of time using the device, information obtained from the device to guide treatment, risk of complications and discreteness.

For peer review only

Participants were asked to select their most preferred option. Finally, there was an optional section at the end of the questionnaire asking participants about their socio-demographic characteristics, with a range of questions informed by those characteristics which have been found to affect fertility/ pregnancy or were hypothesised to influence treatment uptake.

To set up the choice context, participants were asked to imagine that their doctor had given them the option of using a novel intrauterine sensor device to find out more about their womb environment, which may help guide them in making decisions about next treatment steps towards a successful pregnancy. They were asked to choose the preferred device in each choice set if these were offered as investigation options in 'real life'.

**Analysis of data**

We performed econometric analysis from the responses obtained from the DCE questionnaire. Conditional logit models were initially estimated. Unobserved preference heterogeneity was explored through a latent class model (LCM) whereby individual are probabilistically sorted into classes whose number is exogenously set. LCM allows identification of distinct behavioural and preference patterns within the data and different regression parameters are estimated for each class (8, 9). Further, we parameterize the class membership equation to assess whether given individual characteristics explain which individuals fall within which class.

Coefficients in DCE models capture whether an attribute level increases or decreases the attractiveness (or utility in economic terms) of a device featuring such characteristics. However, coefficients (due to variance normalization) lack direct quantitative interpretation. Yet, within each model and across classes the relative importance of coefficients is feasible, while interpretation can also rely on ratios of coefficients.

For peer review only

To provide further, intuitive interpretation of results, we calculate and present predicted probabilities for two sets of eight hypothetical devices with varying characteristics, ranging from the one that would seem to be the most attractive to the least attractive. The resulting probabilities essentially gives the probability that any given device would be selected by respondents if the eight presented where the available options.

**Patient public involvement**

Engagement of patients is critical to the design of the DCE questionnaire and this was introduced from conception of the study. The key attributes and levels were identified through a series of focus groups involving potential users for the novel intrauterine device. A preliminary DCE questionnaire was piloted on 10 women within the Obstetrics and Gynaecology Department. Further interviews with the women refined the questionnaire's comprehensibility, usability, amount of choice sets and content validity of the attributes. Feedback from the PPI work shaped the exact formulation of the attributes and the DCE instructions on final DCE questionnaire.

For peer review only

## Results

### Study participants

361 women were recruited into this DCE questionnaire study. Table 3 summarises the participant characteristics. The mean age of the participants was 32.5 years (SD 5.23). Most of the participants (60.1%) were married, 23.9% were in a relationship, 11% were engaged, and 3.8% were single. Over half of the participants (52.3%) had higher education and professional/vocational equivalents. 81.9% of the women were needing treatment for infertility and 5.4% were needing treatment for recurrent miscarriage. 22.3% had a child (either partner). 30.4% qualified for NHS funded IVF.

| | | **Mean** | **Standard deviation** |
|---|---|---|---|
| **Age** | | 32.5 | 5.23 |
| | | | |
| | | **N** | **%** |
| **Relationship status** | Single | 7 | 3.8 |
| | In a relationship, living apart | 3 | 1.5 |
| | In a relationship, living together | 41 | 22.4 |
| | Engaged | 20 | 11.0 |
| | Married | 110 | 60.1 |
| | Civil partnership | 1 | 0.6 |
| | Living with parents | 1 | 0.6 |
| | | | |
| | | **N** | % |
| **Educational attainment** | Higher education & professional/vocational equivalents | 184 | 52.3 |

For peer review only

| | Conditional Logit | Latent Class Conditional Logit | |
|---|---|---|---|
| | | Class 1 | Class 2 |
| Preferences | | | |
| Length of time (1 day decrease) | 0.0150*** | 0.0111 | 0.0271*** |

| | | | |
|---|---|---|---|
| | A levels, vocational level 3 and equivalents | 81 | 23.0 |
| | GCSE/O level grade A*-C, vocational level 2 and equivalents | 62 | 17.6 |
| | Qualifications at level 1 and below | 9 | 2.6 |
| | Other qualifications: level unknown (including foreign qualifications) | 9 | 2.6 |
| | No qualifications | 7 | 2.0 |
| | | | |
| | | **N** | % |
| **Needing treatment for** | Infertility/subfertility | 289 | 81.9 |
| | Recurrent miscarriages (3 or more consecutive early miscarriages) | 19 | 5.4 |
| | | | |
| | | **N** | % |
| **Have children (either partner)** | | 79 | 22.3 |
| | | | |
| | | **N** | % |
| **Qualify for NHS funded IVF** | Yes | 105 | 30.4 |
| | No | 92 | 26.6 |
| | Unsure | 149 | 43.1 |

**Table 3** Summary of study participants.

**Conditional Logit**

| | | | |
|---|---|---|---|
| | (0.00425) | (0.00682) | (0.00935) |
| Information (base: Limited) | | | |
| Majority | 1.243*** | 1.021*** | 2.008*** |
| | (0.142) | (0.333) | (0.228) |
| All | 2.771*** | 1.996*** | 3.456*** |
| | (0.209) | (0.349) | (0.287) |
| Risk of complications | 0.184*** | 0.0216 | 0.318*** |
| (1% decrease) | (0.00978) | (0.0161) | (0.0206) |
| Discreteness (base: Indiscrete) | | | |
| Completely | 1.104*** | 1.250*** | 1.462*** |
| | (0.0991) | (0.158) | (0.190) |
| Moderately | 0.793*** | 0.807*** | 1.349*** |
| | (0.0905) | (0.164) | (0.222) |
| Class probabilities | | | |
| Higher education | | -0.660** | -- |
| | | (0.307) | |
| Qualify for NHS funded IVF | | -0.709** | -- |
| | | (0.356) | |
| Constant | | -0.463* | -- |
| | | (0.252) | |
| Class Shares | | 0.274 | 0.726 |
| # of Obs | 6,496 | 6,190 | |
| # of Resp | 361 | 344 | |
| BIC | 2639 | 2425 | |

**Table 4** Women's preferences in the four features of the intrauterine monitoring device: length of time using the device (per day), information obtained from the device to guide treatment, risk of complications (per 1% increase) and discreteness. Co-efficients for the conditional logit and latent class conditional logit are shown along with robust standard errors in parentheses; *** p<0.01, ** p<0.05, * p<0.1.

The results of the conditional logit are presented in Table 4. Two-way interactions with the information attribute were not statistically significant and did not reveal significantly different preference patterns. Further, we test the need for alternative-specific constant (i.e. whether respondents for some reasons preferred devices presented first over those presented second, irrespective of characteristics) and confirm its lack of statistical significance. Hence, we focus on main effects models without alternative-specific constants. Finally, using a heteroskedastic logit specification we test for scaling differences between the blocks and again confirm lack of evidence for any differences allowing us to pool and analyse the data.

From the conditional logit co-efficients, it is possible to make relative comparisons in the magnitude of the co-efficients, comparing between the effect of different attributes and attribute levels. Women placed the most importance on the attribute of obtaining information

to guide treatment, followed by discreteness of the device and its receiver, risk of complications and lastly length of time of use.

Having a device that provides information that guides treatment in all cases (co-efficient 2.771) is over two times more attractive as a device which provides information in majority of cases (co-efficient 1.243). Women placed the same level of importance in a device that provides information that guides treatment in all cases (co-efficient 2.771) and a device whereby the risk of complications (co-efficient 0.184 per 1% decrease) is reduced by 15%. Completely discrete devices (co-efficient 1.104) were over 1.5 times more attractive than moderately discrete devices (co-efficient 0.793). Women put the same level of importance on 12 days decrease in the length of use of the device (co-efficient 0.015 per 1 day decrease) as a 1% decrease in the risk of complications from using the device (co-efficient 0.184 per 1% decrease).

**Latent class conditional logit**

The latent class conditional logit analysis probabilistically assigns participants to two classes with 27.4% of participants falling within class 1 and 72.6% within class 2 (Table 4). In general, class 1 participants are less likely to have higher education (co-efficient -0.660, standard error 0.307, 95% CI -0.967 and -0.353), and less likely to qualify for NHS funded IVF (co-efficient -0.709, standard error 0.356, 95% CI -1.065 and -0.353).

*Class 1*

Having a device that provides information that guides treatment in all cases (co-efficient 1.996) is almost two times as attractive as a device which provides information in majority of cases (co-efficient 1.021). Moving from a completely discrete device (co-efficient 1.250) to a moderately discrete device (co-efficient 0.807) reduced the attractiveness by a third. Length

For peer review only

of use of the device and risk of complications are not significant factors altering participant's preference for the device.

*Class 2*

Having a device that provides information that guides treatment in all cases (co-efficient 3.456) is 1.7 times more attractive than a device which provides information in majority of cases (co-efficient 2.008). Women put the same level of importance on 12 days decrease in the length of use of the device (co-efficient 0.0271 per 1 day decrease) as a 1% decrease in the risk of complications from using the device (co-efficient 0.318 per 1% decrease). Obtaining information that guided treatment in all cases (co-efficient 3.456) had an equal importance to an 11% decrease in the risk of complications (co-efficient 0.318 per 1% decrease) .

Obtaining information that guided treatment in all cases (co-efficient 3.456) had an equal importance to a reduction in the length of use (co-efficient 0.0271 per 1 day decrease) by 128 days. There was a slight decrease in the attractiveness of the device when the receiver was moderately discrete (co-efficient 1.349) compared to completely discrete (co-efficient 1.462).

*Comparing Class 1 to Class 2*

Those in class 2 placed more importance on a device which provided information to guide treatment. Compared to the baseline, obtaining information to guide treatment in all cases was 1.7 times more important in class 2 (co-efficient 3.456) compared to class 1 (co-efficient 1.996). Obtaining information to guide treatment in majority of cases was two times more important in class 2 (co-efficient 2.008) compared to class 1 (co-efficient 1.021).

Class 2 participants placed more importance on length of use for the device. A device which has an increased length of use (by 1 day) is nearly 2.5 times less attractive for class 2 (co-efficient 0.0271 per 1 day decrease) than class 1 (co-efficient 0.0111 per 1 day decrease).

For peer review only

Class 2 participants placed more importance on the risk of complications. A device which had a 1% decrease in the risk of complications is nearly 15 times more attractive for class 2 (co-efficient 0.318 per 1% decrease) than class 1 (co-efficient 0.0216 per 1% decrease). Class 2 participants placed a slightly higher importance on having a discrete device. Having a completely discrete device was more attractive for class 2 (co-efficient 1.462) than class 1 (co-efficient 1.250)

**Predicted probabilities**

Given that the results in both the conditional logit and the latent class conditional logit have a similar pattern of preferences, with some differences in the magnitudes between the two classes, we focus on the predicted probabilities of the conditional logit. Predicted probabilities indicate the probability that any one of the 8 hypothetical devices described will be chosen when all 8 are on offer (Table 5). The importance of the combinations of the characteristics of each of these hypothetical devices is represented by the magnitude of their probabilities.

For peer review only

A)

| Hypothetical device | A | B | C | D | E | F | G | H |
|---|---|---|---|---|---|---|---|---|
| Length of time using the device | 7 days | 7 days | 14 days | 7 days | 7 days | 14 days | 7 days | 28 days |
| Information obtained from device and its use in guiding treatment | ALL cases | MAJORITY of cases | MAJORITY of cases | ALL cases | LIMITED cases | LIMITED cases | LIMITED cases | LIMITED cases |
| Risk of complications | 1% | 1% | 1% | 10% | 1% | 1% | 10% | 10% |
| Discreteness of the information receiver | Completely | Completely | Moderately | Moderately | Completely | Moderately | Moderately | Indiscrete |
| **Predicted probabilities (Conditional Logit)** | **61.9%** | **13.4%** | **8.9%** | **8.6%** | **3.9%** | **2.6%** | **0.5%** | **0.2%** |

B)

| Hypothetical device | A | B | C | D | E | F | G | H |
|---|---|---|---|---|---|---|---|---|
| Length of time using the device | 7 days | 14 days | 7 days | 14 days | 7 days | 14 days | 7 days | 14 days |
| Information obtained from device and its use in guiding treatment | LIMITED cases | LIMITED cases | LIMITED cases | LIMITED cases | LIMITED cases | LIMITED cases | LIMITED cases | LIMITED cases |
| Risk of complications | 1% | 1% | 1% | 1% | 10% | 10% | 10% | 10% |
| Discreteness of the information receiver | Completely | Completely | Moderately | Moderately | Completely | Completely | Moderately | Moderately |
| **Predicted probabilities (Conditional Logit)** | **25.5%** | **23.0%** | **18.7%** | **16.8%** | **4.9%** | **4.4%** | **3.6%** | **3.2%** |

**Table 5** Features and predicted probabilities of 8 hypothetical devices (A-F) being chosen according to conditional logit. A) shows various combinations of attribute levels, ranging from the most preferred device to the least preferred device attribute levels. B) shows various combinations of attribute levels of the device, fixing one attribute, information obtained device and its use in guiding treatment to a 'limited' number of cases.

For peer review only

When 8 hypothetical devices are presented to an individual with varying levels of different attributes (Table 5A), over 60% of the time, hypothetical device A will be chosen (shortest length of time using the device (7 days), obtaining information that guided treatment in all cases, lowest risk of complications (1%) and completely discrete). About 13% of the time, they are predicted to choose hypothetical device B (7 days, obtaining information that guided treatment in majority of the cases, 1% risk and completely discrete). About 9% of the time, an individual would choose device D (7 days, obtaining information that guided treatment in all cases, 10% risk and moderately discrete).

Next, we assess the effect on predicted probabilities when the devices presented were all able to provide information to guide treatment in a limited number of cases, with varying levels of the other attributes. When 8 hypothetical devices, with the information attribute fixed to guiding treatment in a limited number of cases are presented (Table 5B), about 25% of the time, an individual would choose Device A (7 days, 1% risk, completely discrete), 23% of the time, they would choose device B (14 days, 1% risk, completely discrete) and 19% of the time, they would choose device C (7 days, 1% risk, moderately discrete). The four devices with the higher risk of complications (10%) had a low likelihood (<5%) of being selected.

For peer review only

## Discussion

To our knowledge, this is the first DCE study of women's preferences for a novel intrauterine monitoring device as an investigative tool designed to monitor the womb environment. It provides insights into the preferred attributes of a novel device, and the influence of sociodemographic characteristics on choices women make. These findings will help with the further development of the monitoring device and to ensure that the women's preferences are considered prior to the design of clinical trials using the device.

We have shown that all four selected attributes played a significant role in women's preferences for the intrauterine monitoring device. The ideal attributes of the device would be that it is used for the shortest length of time, guide treatment in all cases, have the lowest risk of complications, and be completely discrete. In terms of importance of the device attributes the ability of the device to obtain information and guide treatment was most important, followed by discreteness of the device, then risk of complications and finally length of using the device.

Women were willing to have a device for a longer length of time, with higher risk of complications or an indiscrete device if it was able to provide information that guides treatment in all cases. The importance of the device being able to guide treatment is emphasised by the fact that its attractiveness is doubled when the device can guide treatment in all cases, rather than only in a majority of cases. Currently, about 15-20% of couples who are being investigated for infertility and about 50% of couples being investigated for recurrent miscarriage have no known cause for their reproductive problems and are deemed as 'unexplained' after their diagnostic workup (10, 11). This tends to lead to frustration for the couples, and it seems that a device which can guide treatment in all cases of reproductive failures is most preferred. At present, unexplained infertility is managed by offering blanket IVF treatment to couples as current investigative tools are not able to triage the patients into the appropriate disease/treatment groups. In the cohort of patients with unexplained recurrent pregnancy loss, the main emphasis following on from a negative

For peer review only

diagnostic workup is supportive care (12). Our inability to find a cause of the couple's infertility or repeated miscarriages does not mean that there is no cause for the disorder; endometrial receptivity and the uterine environment are key areas which warrant further investigation in search for possible treatable causes and personalised treatment (13). A reproductive investigative tool is much more likely to be opted for if it helps provide answers to help guide treatment.

Discreteness of a fertility assessment tool plays an important role in women's preferences; having a device that is completely discrete is over 1.5 times more attractive than a device that is moderately discrete. This effect is more pronounced in the cohort of women who fall in a category where they are more likely to have higher education and more likely to qualify for NHS funded IVF. Women who have reproductive disorders often feel isolated and stigmatised (14, 15) and thus the value they place on "not being visible" in society is high. When women are affected by infertility, many choose not to share their problem with others including family members because of the stigmatising and repressive perspectives of society towards people who can't conceive naturally and towards IVF treatment (16). Moreover, despite psychological counselling being offered to patients with reproductive disorders, the uptake is low (17). These features tend to support our findings of the infertile women not wanting to be 'highlighted' or 'labelled' by an indiscrete reproductive investigative device in society.

Women valued the importance of having a device which has the lowest risk of complications; they were willing to use the device for almost 2 weeks longer if it was able to lead to a 1% reduction in the risk of complications from the device. Given risk perceptions involve incorporating numerical information about threat, the ability to understand and use the numeric information presented plays a significant role in the formation and use of risk perceptions (18). Individuals who are well educated are more likely to retrieve and use numerical principles in decision making, and within our analyses, we have shown that women who are more likely to fall in the category who have had higher education place more

For peer review only

importance on a device with lower risk of complications (1% reduction in the risk of complications is 15 times more important to the group of women who are more likely to fall within the group who have had higher education).

The length of time using the device does not seem to play a major role in women's preferences towards the use of the novel investigative tool for reproductive disorders. Within our cohort of women, if the device is able to provide information to guide treatment in all cases, they were willing to use the device for up to 184 days.

For peer review only

**Limitations**

Participants were recruited in a single UK hospital, which may limit its applicability in other parts of the UK or in other countries. Future research could compare women's preferences across different populations, cultures and health service provisions (e.g. NHS or private patient cohorts).

Although DCEs have been widely used in many areas of healthcare to elicit patient preferences, they have important limitations. Participants are evaluating the hypothetical choice sets intended to simulate real life decisions, but the responses may not accurately reflect real choices as participants do not experience the resulting consequences of their decisions. However, the fact that results are in line with prior expectations mitigates such concerns. Another limitation is the restricted number of attributes and levels that can be included in the choice sets to reduce cognitive burden and for it to be practically feasible. We have also not included a cost attribute as our aim was to explore the trade-offs between the attributes which seemed most important to users from PPI work that was performed prior to the study, rather than include economic analysis. To quantify trade off in one attribute relative to another, we have assumed that the levels are linear, e.g. the risk of complications is linear between the two levels (1% and 10%); in reality the risk preferences between these levels may not be linear.

## Conclusions

Within women planning a pregnancy, all four selected attributes played a significant role in altering women's preference for a novel intrauterine device designed to monitor the womb environment. Women seemed to place most importance on having a device that obtains information to guide treatment and are willing to use the device for a longer length of time, have a device with higher risk of complications and an indiscrete device if it is able to provide answers and direction for treatment of their reproductive disorder. Women who are more likely to have higher education and qualify for NHS funded IVF place more importance on all attributes.

## Funding

This study has been funded by the NIHR i4i Innovation grant (Grant number II-LB-0715-20002) awarded to Professor Cheong.

## Acknowledgements

The authors would like to thank the Obstetrics and Gynaecology Research Team (with special mention to Susan Wellstead and Agnieszka Burtt) at the Princess Anne Hospital, and the gynaecology outpatient team at the Complete Fertility Unit for their hard work and help with conducting the study. We would also like to extend our gratitude to all the women who took part in the study.

## Author contributions

For peer review only

KYBN and YC conceived the idea. KYBN, EM and YC designed the experiments. KYBN, RE recruited participants into the study. All co-authors analysed and interpreted the data. KYBN RE wrote the initial draft of the paper and the final version was approved by all co-authors prior to submission.

**Competing interests**

Professor Ying Cheong is a co-founder, non-executive Director and minority shareholder of Verso Biosense.

**Data Availability**

Anonymised participant data are available upon reasonable request by contacting the corresponding author.

For peer review only

## References


1. Hanson K, McPake B, Nakamba P, Archard L. Preferences for hospital quality in Zambia: results from a discrete choice experiment. Health Econ. 2005;14(7):687-701.
2. Ryan M. Discrete choice experiments in health care. BMJ. 2004;328(7436):360-1.
3. McIntosh E. Using discrete choice experiments within a cost-benefit analysis framework: some considerations. Pharmacoeconomics. 2006;24(9):855-68.
4. Botha W, Donnolley N, Shanahan M, Norman RJ, Chambers GM. Societal preferences for fertility treatment in Australia: a stated preference discrete choice experiment. J Med Econ. 2019;22(1):95-107.
5. Bech M, Kjaer T, Lauridsen J. Does the number of choice sets matter? Results from a web survey applying a discrete choice experiment. Health Econ. 2011;20(3):273-86.
6. van Empel IW, Dancet EA, Koolman XH, Nelen WL, Stolk EA, Sermeus W, et al. Physicians underestimate the importance of patient-centredness to patients: a discrete choice experiment in fertility care. Hum Reprod. 2011;26(3):584-93.
7. Braam SC, de Bruin JP, Mol BWJ, van Wely M. The perspective of women with an increased risk of OHSS regarding the safety and burden of IVF: a discrete choice experiment. Hum Reprod Open. 2020;2020(2):hoz034.
8. Hensher D, Rose J, Greene W. Applied Choice Analysis: A Primer. Cambridge, UK: Cambridge University Press; 2005.
9. Greene WH, Hensher DA. A latent class model for discrete choice analysis: contrasts with mixed logit. Transport Res B-Meth. 2003;37(8):681-98.
10. Practice Committee of the American Society for Reproductive M. Effectiveness and treatment for unexplained infertility. Fertil Steril. 2006;86(5 Suppl 1):S111-4.
11. Practice Committee of the American Society for Reproductive Medicine. Electronic address aao. Definitions of infertility and recurrent pregnancy loss: a committee opinion. Fertil Steril. 2020;113(3):533-5.
12. ESHRE Early Pregnancy Guideline Development Group. Recurrent pregnancy loss Version 22017.
13. Hatasaka H. New perspectives for unexplained infertility. Clin Obstet Gynecol. 2011;54(4):727-33.
14. Slade P, O'Neill C, Simpson AJ, Lashen H. The relationship between perceived stigma, disclosure patterns, support and distress in new attendees at an infertility clinic. Hum Reprod. 2007;22(8):2309-17.
15. Zhang F, Lv Y, Wang Y, Cheng X, Yan Y, Zhang Y, et al. The social stigma of infertile women in Zhejiang Province, China: a questionnaire-based study. BMC Womens Health. 2021;21(1):97.
16. Sahin BM, Gursoy E. The social and psychological consequences of women getting pregnant with fertility treatment: A qualitative study. Perspect Psychiatr Care. 2021;57(2):463-72.
17. Boivin J, Scanlan LC, Walker SM. Why are infertile patients not using psychosocial counselling? Hum Reprod. 1999;14(5):1384-91.
18. Peters E, Vastfjall D, Slovic P, Mertz CK, Mazzocco K, Dickert S. Numeracy and decision making. Psychol Sci. 2006;17(5):407-13.


For peer review only

**Figure legends**

Figure 1- Pictoral representation of the novel intrauterine device and on how it will sit within the uterus. The sensor is batteryless and is powered using a belt and a mobile phone. A belt worn around the waist delivers energy to the sensor and collects data when required. A smartphone controls the system and collects the data.

For peer review only

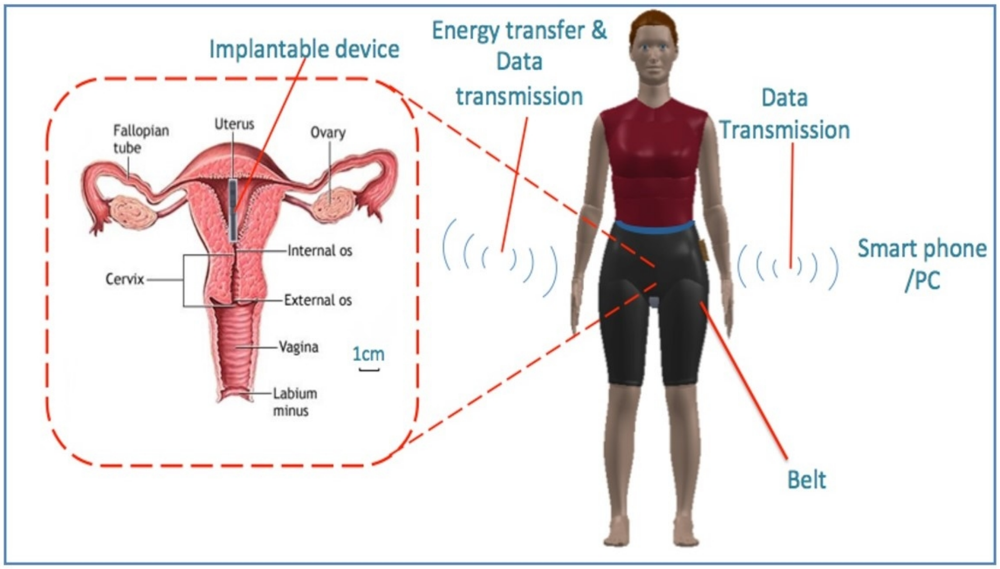


Figure 1- Pictoral representation of the novel intrauterine device and on how it will sit within the uterus. The sensor is batteryless and is powered using a belt and a mobile phone. A belt worn around the waist delivers energy to the sensor and collects data when required. A smartphone controls the system and collects the data.

202x120mm (144 x 144 DPI)

UNIVERSITY OF Southampton VivoPlex NHS University Hospital Southampton NHS Foundation Trust NHS National Institute for Health Research

# Investigations for women with reproductive problems.

# A questionnaire study.

# What are patients' preferences for a novel device able to monitor the womb environment?

## Introduction

Doctors and researchers at the University of Southampton in collaboration with VivoPlex Medical Ltd are developing and carrying out research into a novel monitoring device that may be offered to women with fertility issues. Whilst this device has not yet been produced, we are interested in your views about this new sensor device, and what features would be important to you. Participating in this would involve you completing the attached questionnaire. Once this questionnaire is complete and handed to a member of staff, you are not required to do anything further. Responses will be anonymous and confidential. Your views would help us to develop and tailor this novel sensor for women planning a pregnancy in the future. It is important to understand this device is not yet available to you.

We would like you to *imagine* that your doctor has given you the option of using a sensor device in order to find out more about your womb environment. Once the sensor device is inserted into the womb, you would also be asked to wear a garment close to your womb, for a number of days, that holds a small battery pack and information receiver, all supplied to you along with the sensor. The sensor works by measuring three characteristics of your womb environment continuously, and sending that information back to the receiver. Your doctor would discuss the information with you which may allow them to guide you in making decisions about your next treatment steps towards a successful pregnancy.


For peer review only - http://bmjopen.bmj.com/site/about/guidelines.xhtml

University of Southampton VivoPlex NHS University Hospital Southampton NHS Foundation Trust NHS National Institute for Health Research

## Patient information about the study

### What is the purpose of the study?

This is a questionnaire study to help us understand what women consider the most important features of the device. By completing the following questionnaire you could help us to develop important features of this novel test.

### Why have I been chosen and do I need to take part?

As you are attending the outpatient clinic and hoping to have a baby in the future, we would be interested in your opinions about this novel device. We are asking if you would be prepared to complete this questionnaire. You can decide whether or not to take part, and your decision will have no impact on your care.

### What will happen to me if I decide to take part?

Completing the attached questionnaire will take around 10 minutes, and once completed you should return it to the 'questionnaire collection box' at reception. There will be no follow up after the questionnaire has been handed in, and your answers will be confidential and anonymous, and accessed only by members of the research team. The questionnaire asks questions around sensitive topics including fertility and conception and if you do not feel comfortable answering them, you can withdraw from the study at any time without your clinical care being affected.

### What will happen to the results of the research?

The findings will be presented at scientific meetings, nationally and internationally, published in medical journals, and possibly in the local and national press. You will not be identified in these reports or publications. The University policy for data protection (https://www.southampton.ac.uk/about/governance/policies/privacy-policy.page) will be followed.

### Who is organising and funding the research?

This study is funded by the National Institute for Health Research (NIHR) Invention for Innovation stream which supports clinical development of medical devices in areas of existing or emerging patient need. The study is being led by Professor Ying Cheong from the University of Southampton. This study has been reviewed and approved by X Ethics Committee (Ethics No.: to be confirmed).

### Contact for further information.

For further information please contact Dr Bonnie Ng at the University of Southampton, email: bonnie.ng@soton.ac.uk. Or ask to speak to a member of the reproductive health research team 023 8120 6856


For peer review only - http://bmjopen.bmj.com/site/about/guidelines.xhtml

University of Southampton · VivoPlex · NHS University Hospital Southampton NHS Foundation Trust · NHS National Institute for Health Research

**Information on your data**

The University of Southampton is the sponsor or this study based in the United Kingdom, Southampton. We will be using information from you in order to undertake this study and will act as the data controller for this study. This means that we are responsible for looking after your information and using it properly. The University of Southampton will keep identifiable information about you for 10 years after the study has finished.

Your rights to access, change or move your information are limited, as we need to manage your information in specific ways in order for the research to be reliable and accurate. If you withdraw from the study, we will keep the information about you that we have already obtained. To safeguard your rights we will use the minimum personally identifiable information possible.

You can find more about how we use your information at:

http://www.calendar.soton.ac.uk/sectionIV/research-data-management.html

The University of Southampton will collect information from you for this research study in accordance with our instructions.

The University Hospitals Southampton NHS Foundation Trust will keep your name, and contact details confidential and will not pass this information to the University of Southampton. The University Hospitals Southampton NHS Foundation Trust will use this information as needed, to contact you about the research study, and make sure that relevant information about the study is recorded for your care, and to oversee the quality of the study. Certain individuals from the University of Southampton and regulatory organisations may look at your medical and research records to check the accuracy of the research study. The University of Southampton will only receive information without any identifying information The people who analyse the information will not be able to identify you and will not be able to find your name or contact details.

The University Hospitals Southampton NHS Foundation Trust will keep identifiable information about you from this study for 10 years after the study has finished.

**Please pull off the front 2 pages and keep for future reference**


For peer review only - http://bmjopen.bmj.com/site/about/guidelines.xhtml

University Hospital Southampton NHS Foundation Trust

National Institute for Health Research


For peer review only - http://bmjopen.bmj.com/site/about/guidelines.xhtml

UNIVERSITY OF Southampton · VivoPlex · NHS University Hospital Southampton NHS Foundation Trust · NHS National Institute for Health Research

## CONSENTING TO THE QUESTIONNAIRE.

If you have any further questions please ask a member of staff to contact the research team on 02381206856.

By ticking this box ☐ you confirm that:

- You have read the information at the start of this questionnaire.
- You understand who to contact with any questions.
- You understand participation in completely voluntary.
- You are over 18 and under 50 years old.
- You are attending an outpatients appointment at the Princess Anne Hospital.

**Study ID** ☐

**When you have completed this questionnaire, please return to the 'Questionnaire collection box' at reception.**

**If you have partially completed the questionnaire or prefer not to take part, you can also return your questionnaire in the same box.**

**Thank you for your time**


For peer review only - http://bmjopen.bmj.com/site/about/guidelines.xhtml

UNIVERSITY OF Southampton VivoPlex NHS University Hospital Southampton NHS Foundation Trust NHS National Institute for Health Research

**EXPLANATION ABOUT THE QUESTIONNAIRE.**

In this questionnaire you will be presented with several **hypothetical** scenarios in which two sensors with different characteristics will be described. In each situation, we would like you to choose which sensor you prefer out of two options: Device **A** or Device **B**.

Each sensor will be described to you in terms of four features:

1. **Length of time using the device.** The number of days the device would remain inside your womb. Longer time in your womb could give more information to the doctor. There will be three choices, ***7 days, 14 days, or 28 days***

2. **Information obtained from the device and its use in guiding treatment.** There are three choices, the information obtained will guide treatment in a ***limited*** number of cases, ***majority*** of cases or ***all*** cases.

3. **Risk of complications.** There may either be short- or long-term complications from using the device. The long-term effects of using the device are currently unknown, and further clinical trials will be conducted to explore this. The short-term complications could be an infection needing antibiotic treatment, pain or discomfort needing pain relief or expulsion (device falling out of the womb). There will be two choices, 1 in 100 women (***1%, lower risk***), or 10 in 100 women (***10%, higher risk***).

4. **Discreteness of the information receiver.** Once the sensor is inserted into the womb, you will be asked to wear a garment which holds the information receiver. This receiver is required for the data on the womb environment to be obtained and interpreted by a clinician. There are three options regarding the discreteness if the information receiver that you will be asked to wear- ***completely discrete*** (i.e. even when tight clothing is worn the receiver will not be noticeable to others), ***moderately discrete*** (i.e. the receiver will be noticeable to others when tight clothing is worn), and ***indiscrete*** (i.e. the receiver will be noticeable to others irrespective of the type of clothing worn).


For peer review only - http://bmjopen.bmj.com/site/about/guidelines.xhtml

University of Southampton · VivoPlex · University Hospital Southampton NHS Foundation Trust · National Institute for Health Research

## HOW TO COMPLETE THIS QUESTIONNAIRE.

**There are no right or wrong answers** - we are just interested in your views. Please choose the option that in your opinion is the better device, the one that best matched your priorities.

## EXAMPLE QUESTION

We will now take you through an example question. You will not need to answer the question in this section.

Consider the devices described by the following two sets of features (Device **A** and Device **B**). Please indicate which choice you would prefer by circling the column.

| | **Device A** | **Device B** |
|---|---|---|
| **Length of time using the device** | 7 Days | 28 Days |
| **Information obtained from the device and its use in guiding treatment** | The information obtained will guide treatment in all cases | The information obtained will guide treatment in all cases |
| **Risk of complications** | 10% | 1% |
| **Discreteness of the information receiver** | Moderately discrete | Completely discrete |

In this example, Device A has been selected. The person made the choice based on the features of Device A and Device B, and decided her preferred choice.

## CHOICE SETS


For peer review only - http://bmjopen.bmj.com/site/about/guidelines.xhtml

Please answer **ALL** 9 choice sets and indicate your choice by drawing a circle around your preferred device.

| Choice Set 1 | | |
|---|---|---|
| | **Device A** | **Device B** |
| **Length of using the device** | 7 Days | 28 Days |
| **Information obtained from the device and its use in guiding treatment** | The information obtained will guide treatment in a limited number of cases | The information obtained will guide treatment in a limited number of cases |
| **Risk of complications** | 1% | 10% |
| **Discreteness of the information receiver** | Completely discrete | Indiscrete |

| Choice Set 2 | | |
|---|---|---|
| | **Device A** | **Device B** |
| **Length of using the device** | 28 Days | 7 Days |
| **Information obtained from the device and its use in guiding treatment** | The information obtained will guide treatment in the majority of cases | The information obtained will guide treatment in the majority of cases |
| **Risk of complications** | 10% | 1% |
| **Discreteness of the information receiver** | Completely discrete | Moderately discrete |

| Choice Set 3 | | |
|---|---|---|
| | **Device A** | **Device B** |
| **Length of using the device** | 7 Days | 14 Days |
| **Information obtained from the device and its use in guiding treatment** | The information obtained will guide treatment in a limited number of cases | The information obtained will guide treatment in a limited number of cases |
| **Risk of complications** | 1% | 10% |
| **Discreteness of the information receiver** | Indiscrete | Completely discrete |

| Choice Set 4 | | |
|---|---|---|
| | **Device A** | **Device B** |


For peer review only - http://bmjopen.bmj.com/site/about/guidelines.xhtml

| | | |
|---|---|---|
| **Length of using the device** | 28 Days | 14 Days |
| **Information obtained from the device and its use in guiding treatment** | The information obtained will guide treatment in a limited number of cases | The information obtained will guide treatment in all cases |
| **Risk of complications** | 10% | 10% |
| **Discreteness of the information receiver** | Moderately discrete | Completely discrete |

| Choice Set 5 | | |
|---|---|---|
| | **Device A** | **Device B** |
| **Length of using the device** | 14 Days | 28 Days |
| **Information obtained from the device and its use in guiding treatment** | The information obtained will guide treatment in all cases | The information obtained will guide treatment in all cases |
| **Risk of complications** | 1% | 10% |
| **Discreteness of the information receiver** | Moderately discrete | Completely discrete |

| Choice Set 6 | | |
|---|---|---|
| | **Device A** | **Device B** |
| **Length of using the device** | 7 Days | 28 Days |
| **Information obtained from the device and its use in guiding treatment** | The information obtained will guide treatment in all cases | The information obtained will guide treatment in all cases |
| **Risk of complications** | 10% | 1% |
| **Discreteness of the information receiver** | Moderately discrete | Indiscrete |

For peer review only


For peer review only - http://bmjopen.bmj.com/site/about/guidelines.xhtml

| Choice Set 7 | | |
|---|---|---|
| | **Device A** | **Device B** |
| **Length of using the device** | 14 Days | 28 Days |
| **Information obtained from the device and its use in guiding treatment** | The information obtained will guide treatment in the majority of cases | The information obtained will guide treatment in the majority of cases |
| **Risk of complications** | 10% | 1% |
| **Discreteness of the information receiver** | Indiscrete | Moderately discrete |

| Choice Set 8 | | |
|---|---|---|
| | **Device A** | **Device B** |
| **Length of using the device** | 14 Days | 7 Days |
| **Information obtained from the device and its use in guiding treatment** | The information obtained will guide treatment in a limited number of cases | The information obtained will guide treatment in a limited number of cases |
| **Risk of complications** | 1% | 10% |
| **Discreteness of the information receiver** | Moderately discrete | Completely discrete |

| Choice Set 9 | | |
|---|---|---|
| | **Device A** | **Device B** |
| **Length of using the device** | 14 Days | 14 Days |
| **Information obtained from the device and its use in guiding treatment** | The information obtained will guide treatment in a limited number of cases | The information obtained will guide treatment in all cases |
| **Risk of complications** | 1% | 1% |
| **Discreteness of the information receiver** | Indiscrete | Completely discrete |



For peer review only - http://bmjopen.bmj.com/site/about/guidelines.xhtml

## Section 4: ABOUT YOURSELF

**Age** ☐ **First 4 characters of your postcode** ☐☐☐☐

**Relationship status**

Single ☐
In a relationship and living apart ☐
In a relationship and living together ☐
Engaged ☐
Married ☐
Civil partnership ☐
Other ☐
Please state___________________________

**Education attainment**

Higher education & professional/vocational equivalents ☐
A levels, vocational level 3 and equivalents ☐
GCSE/O level grade A*-C, vocational level 2 and equivalents ☐
Qualifications at level 1 and below ☐
Other qualifications: level unknown (including foreign qualifications) ☐
No qualifications ☐

**What is your ethnic group?**

White ☐
Mixed/multiple ethnic groups ☐
Asian/Asian British ☐
Black/African/Caribbean/Black British ☐
Other Ethnic group ☐
Please state_________________________________

**Are you needing treatment for infertility/subfertility?**.

Yes ☐ No ☐

**Are you needing treatment for recurrent miscarriages (3 or more consecutive early miscarriages)?**

Yes ☐ No ☐

**Do you or your partner have children?**

Yes ☐ No ☐


For peer review only - http://bmjopen.bmj.com/site/about/guidelines.xhtml
For peer review only

UNIVERSITY OF Southampton   VivoPlex   NHS University Hospital Southampton NHS Foundation Trust   NHS National Institute for Health Research

**Have you had any of the following treatment?**

Hysteroscopy (procedure to look inside the womb) ☐

Diagnostic laparoscopy (keyhole procedure to look inside the abdomen only) ☐

Operative laparoscopy (keyhole procedure to look inside the abdomen AND treat pathology) ☐

NHS funded IVF ☐

Privately funded IVF ☐

**Do you qualify for NHS funded IVF?**

Yes ☐ No ☐ Don't know ☐

**Section 5: FEEDBACK (optional)**

**How easy or difficult did you find it to answer these questions?** Please put an 'X' by your response:

| | |
|---|---|
| Very easy | |
| Quite easy | |
| Neither easy nor difficult | |
| Quite difficult | |
| Very difficult | |

**Please write any comments you would like to make about this questionnaire:**

______________________________________________
______________________________________________
______________________________________________
______________________________________________
______________________________________________

**Do you have any ideas on how to improve the questionnaire or the product?**

______________________________________________
______________________________________________
______________________________________________
______________________________________________
______________________________________________

**Thank you for taking the time to complete this questionnaire. Your answers will help us determine how to better develop a novel sensor device designed for women planning a pregnancy.**


For peer review only - http://bmjopen.bmj.com/site/about/guidelines.xhtml

BMJ Open

# A discrete choice experiment exploring women's preferences in a novel device designed to monitor the womb environment and improve our understanding of reproductive disorders



| | |
|---|---|
| Journal: | *BMJ Open* |
| Manuscript ID | bmjopen-2021-058419.R1 |
| Article Type: | Original research |
| Date Submitted by the Author: | 22-Feb-2022 |



| | |
|---|---|
| Complete List of Authors: | Ng, Ka Ying Bonnie; University of Southampton Faculty of Medicine, School of Human Development and Health; Princess Anne Hospital, Department of Obstetrics and Gynaecology<br>Evans, Rhiannon; Salisbury NHS Foundation Trust, Obstetrics and Gynaecology Department<br>Mentzakis, Emmanouil; University of Southampton, Department of Economics<br>Cheong, Ying; University of Southampton Faculty of Medicine, School of Human Development and Health; Princess Anne Hospital, Department of Obstetrics and Gynaecology |



| | |
|---|---|
| <b>Primary Subject Heading</b>: | Obstetrics and gynaecology |
| Secondary Subject Heading: | Patient-centred medicine, Reproductive medicine |
| Keywords: | GYNAECOLOGY, HEALTH ECONOMICS, REPRODUCTIVE MEDICINE, Subfertility < GYNAECOLOGY |
| | |


SCHOLARONE™
Manuscripts

*I, the Submitting Author has the right to grant and does grant on behalf of all authors of the Work (as defined in the below author licence), an exclusive licence and/or a non-exclusive licence for contributions from authors who are: i) UK Crown employees; ii) where BMJ has agreed a CC-BY licence shall apply, and/or iii) in accordance with the terms applicable for US Federal Government officers or employees acting as part of their official duties; on a worldwide, perpetual, irrevocable, royalty-free basis to BMJ Publishing Group Ltd ("BMJ") its licensees and where the relevant Journal is co-owned by BMJ to the co-owners of the Journal, to publish the Work in this journal and any other BMJ products and to exploit all rights, as set out in our licence.*

*The Submitting Author accepts and understands that any supply made under these terms is made by BMJ to the Submitting Author unless you are acting as an employee on behalf of your employer or a postgraduate student of an affiliated institution which is paying any applicable article publishing charge ("APC") for Open Access articles. Where the Submitting Author wishes to make the Work available on an Open Access basis (and intends to pay the relevant APC), the terms of reuse of such Open Access shall be governed by a Creative Commons licence – details of these licences and which Creative Commons licence will apply to this Work are set out in our licence referred to above.*

*Other than as permitted in any relevant BMJ Author's Self Archiving Policies, I confirm this Work has not been accepted for publication elsewhere, is not being considered for publication elsewhere and does not duplicate material already published. I confirm all authors consent to publication of this Work and authorise the granting of this licence.*

For peer review only

# A discrete choice experiment exploring women's preferences in a novel device designed to monitor the womb environment and improve our understanding of reproductive disorders

Ka Ying Bonnie Ng[1,2*] Rhiannon Evans[3] Emmanouil Mentzakis[4] Ying Cheong[1,2]

[1]Faculty of Medicine, University of Southampton, SO17 1BJ

[2]Obstetrics and Gynaecology department, Princess Anne Hospital, Southampton University Hospitals NHS Foundation Trust, Coxford Road, Southampton SO16 5YA

[3]Obstetrics and Gynaecology Department, Salisbury District Hospital, Salisbury NHS Foundation Trust, Odstock Road, Salisbury, SP2 8BJ

[4]Department of Economics, University of Southampton, SO17 1BJ

*Corresponding author

bonnie.ng@doctors.org.uk (BN)

For peer review only

**Abstract**

Objectives

The study aims to determine the relative importance of key attributes of a novel intrauterine device. The device monitors uterine oxygen, pH and temperature in real time with the aim of improving our understanding and treatment of reproductive disorders.

Design

A Discrete Choice Experiment was used to elicit preferences in this novel investigative tool. The attributes and levels used in the choice scenarios were length of time using the device (7, 14 or 28 days), information obtained to guide treatment (limited, majority or all cases), risk of complications (1% or 10%) and discreteness (completely discrete, moderately discrete, or indiscrete).

Setting

Secondary care hospital in Hampshire, UK

Participants

361 women of reproductive age

Primary and secondary outcome measures

Conditional logit and latent class logit regression models to determine the preference for each attribute.

Results

Conditional logit co-efficients allow comparison between attributes; women placed most importance on obtaining information to guide treatment in all cases (2.771), followed by

For peer review only

having a completely discrete device (1.104), reducing risk of complications by 1% (0.184) and decreased length of time by 1 day (0.0150). All co-efficients p<0.01.

Latent class conditional logit assigns participants to two classes with 27.4% in Class 1 who are less likely to have higher education or qualify for NHS funded IVF compared to Class 2. Those in Class 2 placed 1.7 times more importance on a device whose information guided treatment in all cases and a 1% decrease in complications risk was nearly 15 times more attractive.

Conclusions

Women placed most importance on having a device that obtains information to guide treatment and are willing to use the device for a longer, have a device with higher risk of complications and an indiscrete device if it is able to provide answers and direction for treatment of their reproductive disorder.

For peer review only

**Strengths and Limitations of the Study**

- This discrete choice experiment (DCE) explores women's preferences for a novel intrauterine monitoring device designed as an investigative tool in women with reproductive failures.
- DCEs allow for the elicitation of preferences over characteristics and their trade-offs for (not yet in the market) devices in a structured and robust manner.
- Participants were recruited in a single UK hospital, which may limit the applicability in other parts of the UK or in other countries.
- Inherent to DCE, participants do not experience the resulting consequences of their decisions, therefore their responses may not accurately reflect real choices.
- We have not included a cost attribute as our aim was to explore the trade-offs between the attributes which seemed most important to users rather than economic analysis.

For peer review only

## Introduction

The development and introduction of novel technologies, diagnostic tools and therapies in the area of clinical medicine is rapidly increasing. Within reproductive health, we have seen the recent introduction of technological tools such as Ovusense®, skin or vaginal sensors designed to monitor menstrual cycles and track ovulation whilst a woman is trying to conceive and AneVivo, a  novel device that enables in vivo fertilisation to take place in the maternal womb instead of a laboratory incubator for women who are having assisted reproductive technologies (ART). Despite the rapid development of novel diagnostic and therapeutic tools, including medical devices to assist with conception and pregnancy, there is limited data exploring user's preferences. Engagement of users in the development of novel devices is lacking yet hugely important to inform scientists, researchers, clinicians and policy makers. Most importantly, engagement of users in the development of novel therapies and devices would inform and benefit the users themselves.

Within health economics, Discrete Choice Experiment (DCE) is a quantitative technique used for eliciting individual preferences allowing researchers to explore how individual's value attributes of a programme, product or service. DCEs proceed by asking individuals to state their choice over different hypothetical alternatives over a series of scenarios. It is used increasingly in health services research, primarily to assess patient-stated preferences and willingness to pay for different models of health service delivery (1-3). Within the area of reproductive medicine, DCEs have been used to elicit the preferences placed on fertility treatment (4), including the value of patient centred care (5, 6) and willingness to accept risk and complications of fertility treatment (7).

Recently, an implantable batteryless sensor, which can be placed within the uterus to measure oxygen, pH and temperature has been developed in collaboration with a technology company (Vivoplex, Patent number PCT/GB2017/050609) and University of Southampton (UoS) (Figure 1).

For peer review only

| Attributes | Description | Levels |
|---|---|---|
| **Length of time using the device** | The number of days the device would remain inside your womb. Longer time in your womb could give more information to the doctor. There are 3 choices. | 7 days |
| | | 14 days |
| | | 28 days |
| **Information obtained from the device and its use in guiding treatment** | There are three choices, the information obtained will guide treatment in a ***limited*** number of cases, ***majority*** of cases or ***all*** cases. | Limited number of cases= the device would be able to gather information to guide treatment for some women with a reproductive disorder |
| | | Majority of cases= the device would be able to gather information to guide treatment for most women with a reproductive disorder |
| | | All cases= the device would be able to gather information to guide treatment for anyone who uses it regardless of their reproductive condition |
| **Risk of complications** | There may either be short- or long-term complications from using the device. The long-term effects of using the device are currently unknown, and further clinical trials will be conducted to explore this. The short-term complications could be an infection needing antibiotic treatment, pain or discomfort needing pain relief or expulsion (device falling out of the womb). There will be two choices. | 1% Lower risk |
| | | 10% Higher risk |
| **Discreteness of the information receiver** | Once the sensor is inserted into the womb, you will be asked to wear a garment which holds the information receiver. There are three | Completely discrete= even when tight clothing is worn the receiver will not be noticeable to others |
| | | Moderately discrete= the receiver will be noticeable to others when |

For peer review only

| | | |
|---|---|---|
| | options | tight clothing is worn |
| | | Indiscrete= the receiver will be noticeable to others irrespective of the type of clothing worn |

**Table 1** Attributes and levels for the novel intrauterine monitoring device.

The device is similar in shape and size to a contraceptive coil and has an accompanying wearable garment with an information receiver to collect data. This device has the potential to help personalise and streamline care for women with reproductive disorders such as infertility or repeated miscarriages. Current treatment for women with reproductive disorders remains suboptimal and there is no ability to triage patients into appropriate disease/treatment groups. Up to 20% of couples with infertility and 50% of women with recurrent miscarriages have no identified cause for the reproductive problem and as such are then offered either blanket IVF treatment or supportive care. Additionally, IVF treatment is not always successful; the average birth rate per embryo transferred is 24% in 2018 (8). Endometrial receptivity and the uterine microenvironment are key areas which warrant further investigation to advance the care for women in the field of reproductive medicine.

A number of key attributes for the reproductive device including availability of the device, safety, effectiveness as an investigative tool and inconvenience were previously identified from early Patient and Public Involvement (PPI) work. However, there remains a need to understand what value potential users place on such key attributes, and to explore the relative importance of each attribute if this novel intrauterine device is to be offered as an investigative tool for women affected by reproductive failures in the future.

The aim of this study is to elicit patient preferences for the attributes/characteristics of a novel intrauterine monitoring device as an investigative tool in women with reproductive failures by using a DCE. Insight into the value that women place on key attributes can assist with the further design or development of the device, and the design of clinical trials using

For peer review only

the device. The findings may also guide researchers and clinicians designing novel devices within the area of reproductive medicine.

For peer review only

## Methods

### Study Design

This study used a Discrete Choice Experiment (DCE) to elicit patient preferences regarding a novel intrauterine investigative tool to help them with the treatment of their infertility/recurrent miscarriages.

### Development of attributes and levels

Each hypothetical sensor was be described to the participant in terms of four attributes ((1) length of time using the device, (2) information obtained from the device and its use in guiding treatment, (3) risk of complications and (4) discreteness of the information receiver) summarised in Table 1. These were derived from a Public and Patient Involvement (PPI) session, a literature search and discussion with experts (3 clinicians and researchers working within reproductive medicine and 1 health economist) as to which attributes would be of importance. The attributes and DCE questionnaire were further refined through preliminary testing of the questionnaire with potential users of the device.

### Discrete choice experiment design

The combination of attributes and levels resulted in 54 (3x3x2x3) possible scenarios. However, given cognitive burden and fatigue for the respondents, the number of questions to be asked had to be reduced and hence we opted for a fractional factorial design. For this study we were interested in estimating effects for four attributes as well as all two-way interactions with the information attribute. As such the number of estimable parameters to be identified is 18 (i.e. 17 plus 1 for the constant). A D-efficient fractional factorial design with 18 pair-wise choice-sets was created in SAS software. Literature suggests that 18 choices per

For peer review only

individual is possibly more than respondents can handle, potentially impacting the quality of responses (5). To reduce cognitive burden, the design was blocked into two blocks of 9 choice-sets each. At the end of the experiment a series of questions on patients' demographic and medical characteristics was included.

The preliminary DCE questionnaire was piloted on 10 women within the Obstetrics and Gynaecology Department. The women were interviewed; the questionnaire's comprehensibility, usability, amount of choice sets and content validity of the attributes and levels were discussed. After the pilot testing, changes were implemented and repeat testing was carried out. Feedback from the pilot testing shaped the exact formulation of the attributes and the DCE instructions on final DCE questionnaire (Appendix 1).

**Recruitment of participants**

Participants were women of reproductive age (18 to 50 years), who were wishing to achieve a pregnancy now or sometime in the future. We have included all women and have not limited inclusion of women with reproductive disorders as in future clinical studies, we plan to trial the device in women without reproductive disorders as well as those with a range of reproductive problems in order to determine the uterine environment in physiological as well as pathological conditions. Exclusion criteria included those with limited English or limited ability to understand the study design and questionnaire (e.g. language barrier, learning disability, inability to process the information to complete the questionnaire). Participants who met the inclusion and exclusion criteria were recruited from gynaecology clinics within Princess Anne Hospital, Southampton. Women who agree to participate in the study were asked to complete the DCE questionnaire pack (hard copy).

Precise power calculation for DCEs have not been developed but some rule-of-thumb approaches are available (9). The first suggest that there are at least 50 respondents per

For peer review only

alternative, i.e. 100 for our design. The second, posits that sample size should be larger than 500*c/(t * a), where c is the largest number of levels of attribute (or levels of interaction), t is the number of choice tasks and a is the number of alternatives. Hence, for main effects only estimation we would need around 42 individuals per block (i.e. 84 in total), while for main plus our specified two-way interactions we would need around 125 individuals per block (i.e. 250 in total).

**Data collection**

The study was approved by HRA and Health and Care Research Wales (HCRW) (Research Ethics Committee reference 19/IE08/0036). The choice sets were administered in an anonymous hard copy of the questionnaire pack (Appendix 1). The questionnaire pack consisted of several sections including participant information about the study, consenting to the questionnaire, explanation about the DCE questionnaire and how to complete it, an example question, choice sets, 'about yourself' and an optional feedback section. Within the 'explanation about the questionnaire' section, the attributes and levels were explained. Each choice set consisted of two novel intrauterine device alternatives described according to the four attributes in the format as in table 2. The participants completed the questionnaire themselves and were given oral clarification where required. Each participant was assigned a study ID.

| | **Device A** | **Device B** |
|---|---|---|
| **Length of time using the device** | **7** Days | **28** Days |
| **Information guides treatment** | **All** women | **All** women |
| **Risk of complications** | **10%** | **1%** |
| **Discreteness** | **Indiscrete** | **Moderately discrete** |

**Table 2** An example choice set presented to participants. Within each choice set, participants are asked to consider the devices described by the two sets of attributes and choose between device A or device B. Each hypothetical device presented will vary in the

For peer review only

four attirbutes: length of time using the device, information obtained from the device to guide treatment, risk of complications and discreteness.

Participants were asked to select their most preferred option. Finally, there was an optional section at the end of the questionnaire asking participants about their socio-demographic characteristics, with a range of questions informed by those characteristics which have been found to affect fertility/ pregnancy or were hypothesised to influence treatment uptake.

To set up the choice context, participants were asked to imagine that their doctor had given them the option of using a novel intrauterine sensor device to find out more about their womb environment, which may help guide them in making decisions about next treatment steps towards a successful pregnancy. They were asked to choose the preferred device in each choice set if these were offered as investigation options in 'real life'.

**Analysis of data**

We performed econometric analysis from the responses obtained from the DCE questionnaire. Conditional logit models were initially estimated. Unobserved preference heterogeneity was explored through a latent class model (LCM) whereby individual are probabilistically sorted into classes whose number is exogenously set. LCM allows identification of distinct behavioural and preference patterns within the data and different regression parameters are estimated for each class (9, 10). Further, we parameterize the class membership equation to assess whether given individual characteristics explain which individuals fall within which class.

Coefficients in DCE models capture whether an attribute level increases or decreases the attractiveness (or utility in economic terms) of a device featuring such characteristics. However, coefficients (due to variance normalization) lack direct quantitative interpretation. Yet, within each model and across classes the relative importance of coefficients is feasible, while interpretation can also rely on ratios of coefficients.

For peer review only

To provide further, intuitive interpretation of results, we calculate and present predicted probabilities for two sets of eight hypothetical devices with varying characteristics, ranging from the one that would seem to be the most attractive to the least attractive. The resulting probabilities essentially gives the probability that any given device would be selected by respondents if the eight presented where the available options.

**Patient public involvement**

Engagement of patients is critical to the design of the DCE questionnaire, and this was introduced from conception of the study. The key attributes and levels were identified through a series of focus groups involving potential users for the novel intrauterine device. In the focus groups, women with reproductive failures (subfertility or repeated miscarriages) were asked for their views of the device and their potential involvement in a clinical trial using the device.

A preliminary DCE questionnaire was piloted on 10 women within the Obstetrics and Gynaecology Department. Further interviews with the women refined the questionnaire's comprehensibility, usability, amount of choice sets and content validity of the attributes. Feedback from the PPI work shaped the exact formulation of the attributes and the DCE instructions on final DCE questionnaire.

For peer review only

## Results

### Study participants

361 women were recruited into this DCE questionnaire study. Table 3 summarises the participant characteristics. The mean age of the participants was 32.5 years (SD 5.23). Most of the participants (60.1%) were married, 23.9% were in a relationship, 11% were engaged, and 3.8% were single. Over half of the participants (52.3%) had higher education and professional/vocational equivalents. 90.6% of participants were British, 4.7% were Asian or Asian British, 1.7% were of mixed ethnicity, and 0.8% were Black/African/Caribbean or Black British. 81.9% of the women were needing treatment for infertility and 5.4% were needing treatment for recurrent miscarriage. 22.3% of participants or their partner had already had child. 30.4% qualified for NHS funded IVF.

| | | **Mean** | **Standard deviation** |
|---|---|---|---|
| **Age** | | 32.5 | 5.23 |
| | | | |
| | | **N** | **%** |
| **Relationship status** | Single | 7 | 3.8 |
| | In a relationship, living apart | 3 | 1.5 |
| | In a relationship, living together | 41 | 22.4 |
| | Engaged | 20 | 11.0 |
| | Married | 110 | 60.1 |
| | Civil partnership | 1 | 0.6 |
| | Living with parents | 1 | 0.6 |
| | | | |
| | | **N** | % |

For peer review only

| | | | |
|---|---|---|---|
| **Educational attainment** | Higher education & professional/vocational equivalents | 184 | 52.3 |
| | A levels, vocational level 3 and equivalents | 81 | 23.0 |
| | GCSE/O level grade A*-C, vocational level 2 and equivalents | 62 | 17.6 |
| | Qualifications at level 1 and below | 9 | 2.6 |
| | Other qualifications: level unknown (including foreign qualifications) | 9 | 2.6 |
| | No qualifications | 7 | 2.0 |
| | | | |
| | | **N** | % |
| **Ethnicity** | British | 327 | 90.6 |
| | Asian/Asian British | 17 | 4.7 |
| | Mixed ethnicity | 6 | 1.7 |
| | Black/African/Caribbean/Black British | 3 | 0.8 |
| | Other | 3 | 0.8 |
| | | | |
| | | **N** | % |
| **Needing treatment for** | Infertility/subfertility | 289 | 81.9 |
| | Recurrent miscarriages (3 or more consecutive early miscarriages) | 19 | 5.4 |
| | | | |
| | | **N** | % |
| **Either the female participant or her partner have a child** | | 79 | 22.3 |
| | | | |
| | | **N** | % |
| **Qualify for NHS** | Yes | 105 | 30.4 |

For peer review only

| | | | |
|---|---|---|---|
| **funded IVF** | | | |
| | No | 92 | 26.6 |
| | Unsure | 149 | 43.1 |

**Table 3** Summary of study participants.

For peer review only

**Conditional Logit**

| Preferences | Conditional Logit | Latent Class Conditional Logit: Class 1 | Latent Class Conditional Logit: Class 2 |
|---|---|---|---|
| **Length of time (1 day decrease)** | 0.0150***<br>(0.00425) | 0.0111<br>(0.00682) | 0.0271***<br>(0.00935) |
| **Information (baseline: Limited)** | | | |
| Majority | 1.243***<br>(0.142) | 1.021***<br>(0.333) | 2.008***<br>(0.228) |
| All | 2.771***<br>(0.209) | 1.996***<br>(0.349) | 3.456***<br>(0.287) |
| **Risk of complications (1% decrease)** | 0.184***<br>(0.00978) | 0.0216<br>(0.0161) | 0.318***<br>(0.0206) |
| **Discreteness (baseline: Indiscrete)** | | | |
| Completely | 1.104***<br>(0.0991) | 1.250***<br>(0.158) | 1.462***<br>(0.190) |
| Moderately | 0.793***<br>(0.0905) | 0.807***<br>(0.164) | 1.349***<br>(0.222) |
| Class probabilities | | | |
| Higher education | | -0.660**<br>(0.307) | -- |
| Qualify for NHS funded IVF | | -0.709**<br>(0.356) | -- |
| Constant | | -0.463*<br>(0.252) | -- |
| Class Shares | | 0.274 | 0.726 |
| # of Obs | 6,496 | 6,190 | |
| # of Resp | 361 | 344 | |
| BIC | 2639 | 2425 | |

**Table 4** Women's preferences in the four features of the intrauterine monitoring device: length of time using the device (per day), information obtained from the device to guide treatment, risk of complications (per 1% increase) and discreteness. Co-efficients for the conditional logit and latent class conditional logit are shown along with robust standard errors in parentheses; *** p<0.01, ** p<0.05, * p<0.1.

The results of the conditional logit are presented in Table 4. Two-way interactions with the information attribute were not statistically significant and did not reveal significantly different preference patterns. Further, we test the need for alternative-specific constant (i.e. whether respondents for some reasons preferred devices presented first over those presented second, irrespective of characteristics) and confirm its lack of statistical significance. Hence, we focus on main effects models without alternative-specific constants. Finally, using a

For peer review only

heteroskedastic logit specification we test for scaling differences between the blocks and again confirm lack of evidence for any differences allowing us to pool and analyse the data.

From the conditional logit coefficients, it is possible to make relative comparisons in the magnitude of the coefficients, comparing between the effect of different attributes and attribute levels. Women placed the most importance on the attribute of obtaining information to guide treatment, followed by discreteness of the device and its receiver, risk of complications and lastly length of time of use.

Having a device that provides information that guides treatment in all cases (coefficient 2.771) is over two times more attractive as a device which provides information in majority of cases (coefficient 1.243). Women placed the same level of importance in a device that provides information that guides treatment in all cases (coefficient 2.771) and a device whereby the risk of complications (coefficient 0.184 per 1% decrease) is reduced by 15%. Completely discrete devices (coefficient 1.104) were over 1.5 times more attractive than moderately discrete devices (coefficient 0.793). Women put the same level of importance on 12 days decrease in the length of use of the device (coefficient 0.015 per 1 day decrease) as a 1% decrease in the risk of complications from using the device (coefficient 0.184 per 1% decrease).

**Latent class conditional logit**

The latent class conditional logit analysis probabilistically assigns participants to two classes with 27.4% of participants falling within class 1 and 72.6% within class 2 (Table 4). In general, class 1 participants are less likely to have higher education (coefficient -0.660, standard error 0.307, 95% CI -0.967 and -0.353), and less likely to qualify for NHS funded IVF (coefficient -0.709, standard error 0.356, 95% CI -1.065 and -0.353).

*Class 1*

Having a device that provides information that guides treatment in all cases (coefficient 1.996) is almost two times as attractive as a device which provides information in majority of cases (coefficient 1.021). Moving from a completely discrete device (coefficient 1.250) to a moderately discrete device (coefficient 0.807) reduced the attractiveness by a third. Length of use of the device and risk of complications are not significant factors altering participant's preference for the device.

*Class 2*

Having a device that provides information that guides treatment in all cases (coefficient 3.456) is 1.7 times more attractive than a device which provides information in majority of cases (coefficient 2.008). Women put the same level of importance on 12 days decrease in the length of use of the device (coefficient 0.0271 per 1 day decrease) as a 1% decrease in the risk of complications from using the device (coefficient 0.318 per 1% decrease). Obtaining information that guided treatment in all cases (co-efficient 3.456) had an equal importance to an 11% decrease in the risk of complications (coefficient 0.318 per 1% decrease) .

Obtaining information that guided treatment in all cases (coefficient 3.456) had an equal importance to a reduction in the length of use (coefficient 0.0271 per 1 day decrease) by 128 days. There was a slight decrease in the attractiveness of the device when the receiver was moderately discrete (coefficient 1.349) compared to completely discrete (coefficient 1.462).

*Comparing Class 1 to Class 2*

Those in class 2 placed more importance on a device which provided information to guide treatment. Compared to the baseline, obtaining information to guide treatment in all cases was 1.7 times more important in class 2 (coefficient 3.456) compared to class 1 (coefficient 1.996). Obtaining information to guide treatment in majority of cases was two times more important in class 2 (coefficient 2.008) compared to class 1 (coefficient 1.021).

For peer review only

Class 2 participants placed more importance on length of use for the device. A device which has an increased length of use (by 1 day) is nearly 2.5 times less attractive for class 2 (coefficient 0.0271 per 1 day decrease) than class 1 (coefficient 0.0111 per 1 day decrease). Class 2 participants placed more importance on the risk of complications. A device which had a 1% decrease in the risk of complications is nearly 15 times more attractive for class 2 (coefficient 0.318 per 1% decrease) than class 1 (coefficient 0.0216 per 1% decrease). Class 2 participants placed a slightly higher importance on having a discrete device. Having a completely discrete device was more attractive for class 2 (coefficient 1.462) than class 1 (coefficient 1.250).

**Predicted probabilities**

Given that the results in both the conditional logit and the latent class conditional logit have a similar pattern of preferences, with some differences in the magnitudes between the two classes, we focus on the predicted probabilities of the conditional logit. Predicted probabilities indicate the probability that any one of the 8 hypothetical devices described will be chosen when all 8 are on offer (Table 5). The importance of the combinations of the characteristics of each of these hypothetical devices is represented by the magnitude of their probabilities.

For peer review only

A)

| Hypothetical device | A | B | C | D | E | F | G | H |
|---|---|---|---|---|---|---|---|---|
| Length of time using the device | 7 days | 7 days | 14 days | 7 days | 7 days | 14 days | 7 days | 28 days |
| Information obtained from device and its use in guiding treatment | ALL cases | MAJORITY of cases | MAJORITY of cases | ALL cases | LIMITED cases | LIMITED cases | LIMITED cases | LIMITED cases |
| Risk of complications | 1% | 1% | 1% | 10% | 1% | 1% | 10% | 10% |
| Discreteness of the information receiver | Completely | Completely | Moderately | Moderately | Completely | Moderately | Moderately | Indiscrete |
| **Predicted probabilities (Conditional Logit)** | **61.9%** | **13.4%** | **8.9%** | **8.6%** | **3.9%** | **2.6%** | **0.5%** | **0.2%** |

B)

| Hypothetical device | A | B | C | D | E | F | G | H |
|---|---|---|---|---|---|---|---|---|
| Length of time using the device | 7 days | 14 days | 7 days | 14 days | 7 days | 14 days | 7 days | 14 days |
| Information obtained from device and its use in guiding treatment | LIMITED cases | LIMITED cases | LIMITED cases | LIMITED cases | LIMITED cases | LIMITED cases | LIMITED cases | LIMITED cases |
| Risk of complications | 1% | 1% | 1% | 1% | 10% | 10% | 10% | 10% |
| Discreteness of the information receiver | Completely | Completely | Moderately | Moderately | Completely | Completely | Moderately | Moderately |
| **Predicted probabilities (Conditional Logit)** | **25.5%** | **23.0%** | **18.7%** | **16.8%** | **4.9%** | **4.4%** | **3.6%** | **3.2%** |

**Table 5** Features and predicted probabilities of 8 hypothetical devices (A-F) being chosen according to conditional logit. A) shows various combinations of attribute levels, ranging from the most preferred device to the least preferred device attribute levels. B) shows various combinations of attribute levels of the device, fixing one attribute, information obtained device and its use in guiding treatment to a 'limited' number of cases.

For peer review only

When 8 hypothetical devices are presented to an individual with varying levels of different attributes (Table 5A), over 60% of the time, hypothetical device A will be chosen (shortest length of time using the device (7 days), obtaining information that guided treatment in all cases, lowest risk of complications (1%) and completely discrete). About 13% of the time, they are predicted to choose hypothetical device B (7 days, obtaining information that guided treatment in majority of the cases, 1% risk and completely discrete). About 9% of the time, an individual would choose device D (7 days, obtaining information that guided treatment in all cases, 10% risk and moderately discrete).

Next, we assess the effect on predicted probabilities when the devices presented were all able to provide information to guide treatment in a limited number of cases, with varying levels of the other attributes. When 8 hypothetical devices, with the information attribute fixed to guiding treatment in a limited number of cases are presented (Table 5B), about 25% of the time, an individual would choose Device A (7 days, 1% risk, completely discrete), 23% of the time, they would choose device B (14 days, 1% risk, completely discrete) and 19% of the time, they would choose device C (7 days, 1% risk, moderately discrete). The four devices with the higher risk of complications (10%) had a low likelihood (<5%) of being selected.

For peer review only

## Discussion

To our knowledge, this is the first DCE study of women's preferences for a novel intrauterine monitoring device as an investigative tool designed to monitor the womb environment. We have shown that all four selected attributes played a significant role in women's preferences for the intrauterine monitoring device. The ideal attributes of the device would be that it is used for the shortest length of time, guide treatment in all cases, have the lowest risk of complications, and be completely discrete. In terms of importance of the device attributes the ability of the device to obtain information and guide treatment was most important, followed by discreteness of the device, then risk of complications and finally length of using the device. Our study provides insights into the preferred attributes of a novel device, and the influence of sociodemographic characteristics on choices women make. These findings will help with the further development of the monitoring device and to ensure that the women's preferences are considered prior to the design of clinical trials using the device.

Women were willing to have a device for a longer length of time, with higher risk of complications or an indiscrete device if it was able to provide information that guides treatment in all cases. The importance of the device being able to guide treatment is emphasised by the fact that its attractiveness is doubled when the device can guide treatment in all cases, rather than only in a majority of cases. Currently, about 15-20% of couples who are being investigated for infertility and about 50% of couples being investigated for recurrent miscarriage have no known cause for their reproductive problems and are deemed as 'unexplained' after their diagnostic workup (11, 12). This tends to lead to frustration for the couples, and it seems that a device which can guide treatment in all cases of reproductive failures is most preferred. At present, unexplained infertility is managed by offering blanket IVF treatment to couples as current investigative tools are not able to triage the patients into the appropriate disease/treatment groups. In the cohort of patients with unexplained recurrent pregnancy loss, the main emphasis following on from a negative diagnostic workup is supportive care (13). Our inability to find a cause of the couple's

For peer review only

infertility or repeated miscarriages does not mean that there is no cause for the disorder; endometrial receptivity and the uterine environment are key areas which warrant further investigation in search for possible treatable causes and personalised treatment (14). A reproductive investigative tool is much more likely to be opted for if it helps provide answers to help guide treatment.

Discreteness of a fertility assessment tool plays an important role in women's preferences; having a device that is completely discrete is over 1.5 times more attractive than a device that is moderately discrete. This effect is more pronounced in the cohort of women who fall in a category where they are more likely to have higher education and more likely to qualify for NHS funded IVF. Women who have reproductive disorders often feel isolated and stigmatised (15, 16) and thus the value they place on "not being visible" in society is high. When women are affected by infertility, many choose not to share their problem with others including family members because of the stigmatising and repressive perspectives of society towards people who can't conceive naturally and towards IVF treatment (17). Moreover, despite psychological counselling being offered to patients with reproductive disorders, the uptake is low (18). These features tend to support our findings of the infertile women not wanting to be 'highlighted' or 'labelled' by an indiscrete reproductive investigative device in society.

Women valued the importance of having a device which has the lowest risk of complications; they were willing to use the device for almost 2 weeks longer if it was able to lead to a 1% reduction in the risk of complications from the device. Given risk perceptions involve incorporating numerical information about threat, the ability to understand and use the numeric information presented plays a significant role in the formation and use of risk perceptions (19). Individuals who are well educated are more likely to retrieve and use numerical principles in decision making, and within our analyses, we have shown that women who are more likely to fall in the category who have had higher education place more importance on a device with lower risk of complications (1% reduction in the risk of

For peer review only

complications is 15 times more important to the group of women who are more likely to fall within the group who have had higher education).

The length of time using the device does not seem to play a major role in women's preferences towards the use of the novel investigative tool for reproductive disorders. Within our cohort of women, if the device is able to provide information to guide treatment in all cases, they were willing to use the device for up to 184 days.

**Limitations**

Participants were recruited in a single UK hospital, which may limit its applicability in other parts of the UK or in other countries. Future research could compare women's preferences across different populations, cultures and health service provisions (e.g. NHS or private patient cohorts).

Although DCEs have been widely used in many areas of healthcare to elicit patient preferences, they have important limitations. Participants are evaluating the hypothetical choice sets intended to simulate real life decisions, but the responses may not accurately reflect real choices as participants do not experience the resulting consequences of their decisions. However, the fact that results are in line with prior expectations mitigates such concerns. Another limitation is the restricted number of attributes and levels that can be included in the choice sets to reduce cognitive burden and for it to be practically feasible. We have also not included a cost attribute as our aim was to explore the trade-offs between the attributes which seemed most important to users from PPI work that was performed prior to the study, rather than include economic analysis. To quantify trade off in one attribute relative to another, we have assumed that the levels are linear, e.g. the risk of complications is linear between the two levels (1% and 10%); in reality the risk preferences between these levels may not be linear.

For peer review only

### Conclusions

Within women planning a pregnancy, all four selected attributes played a significant role in altering women's preference for a novel intrauterine device designed to monitor the womb environment. Women seemed to place most importance on having a device that obtains information to guide treatment and are willing to use the device for a longer length of time, have a device with higher risk of complications and an indiscrete device if it is able to provide answers and direction for treatment of their reproductive disorder. Women who are more likely to have higher education and qualify for NHS funded IVF place more importance on all attributes.

### Funding

This study has been funded by the NIHR i4i Innovation grant (Grant number II-LB-0715-20002) awarded to Professor Cheong.

### Acknowledgements

The authors would like to thank the Obstetrics and Gynaecology Research Team (with special mention to Susan Wellstead and Agnieszka Burtt) at the Princess Anne Hospital, and the gynaecology outpatient team at the Complete Fertility Unit for their hard work and help with conducting the study. We would also like to extend our gratitude to all the women who took part in the study.

### Author contributions

KYBN and YC conceived the idea. KYBN, EM and YC designed the experiments. KYBN, RE recruited participants into the study. All co-authors analysed and interpreted the data. KYBN

For peer review only

RE wrote the initial draft of the paper and the final version was approved by all co-authors prior to submission.

**Competing interests**

Professor Ying Cheong is a co-founder, non-executive Director and minority shareholder of Verso Biosense.

**Data Availability**

Anonymised participant data are available upon reasonable request by contacting the corresponding author.

For peer review only

## References

1. Hanson K, McPake B, Nakamba P, Archard L. Preferences for hospital quality in Zambia: results from a discrete choice experiment. Health Econ. 2005;14(7):687-701.
2. Ryan M. Discrete choice experiments in health care. BMJ. 2004;328(7436):360-1.
3. McIntosh E. Using discrete choice experiments within a cost-benefit analysis framework: some considerations. Pharmacoeconomics. 2006;24(9):855-68.
4. Botha W, Donnolley N, Shanahan M, Norman RJ, Chambers GM. Societal preferences for fertility treatment in Australia: a stated preference discrete choice experiment. J Med Econ. 2019;22(1):95-107.
5. Bech M, Kjaer T, Lauridsen J. Does the number of choice sets matter? Results from a web survey applying a discrete choice experiment. Health Econ. 2011;20(3):273-86.
6. van Empel IW, Dancet EA, Koolman XH, Nelen WL, Stolk EA, Sermeus W, et al. Physicians underestimate the importance of patient-centredness to patients: a discrete choice experiment in fertility care. Hum Reprod. 2011;26(3):584-93.
7. Braam SC, de Bruin JP, Mol BWJ, van Wely M. The perspective of women with an increased risk of OHSS regarding the safety and burden of IVF: a discrete choice experiment. Hum Reprod Open. 2020;2020(2):hoz034.
8. Human Fertilisation and Embryology Authority. Fertility treatment 2019: trends and figures 2019 [Available from: https://www.hfea.gov.uk/about-us/publications/research-and-data/fertility-treatment-2019-trends-and-figures/#Section1.
9. Hensher D, Rose J, Greene W. Applied Choice Analysis: A Primer. Cambridge, UK: Cambridge University Press; 2005.
10. Greene WH, Hensher DA. A latent class model for discrete choice analysis: contrasts with mixed logit. Transportation Research Part B-Methodological. 2003;37(8):681-98.
11. Practice Committee of the American Society for Reproductive M. Effectiveness and treatment for unexplained infertility. Fertil Steril. 2006;86(5 Suppl 1):S111-4.
12. Practice Committee of the American Society for Reproductive Medicine. Electronic address aao. Definitions of infertility and recurrent pregnancy loss: a committee opinion. Fertil Steril. 2020;113(3):533-5.
13. ESHRE Early Pregnancy Guideline Development Group. Recurrent pregnancy loss Version 22017.
14. Hatasaka H. New perspectives for unexplained infertility. Clin Obstet Gynecol. 2011;54(4):727-33.
15. Slade P, O'Neill C, Simpson AJ, Lashen H. The relationship between perceived stigma, disclosure patterns, support and distress in new attendees at an infertility clinic. Hum Reprod. 2007;22(8):2309-17.
16. Zhang F, Lv Y, Wang Y, Cheng X, Yan Y, Zhang Y, et al. The social stigma of infertile women in Zhejiang Province, China: a questionnaire-based study. BMC Womens Health. 2021;21(1):97.
17. Sahin BM, Gursoy E. The social and psychological consequences of women getting pregnant with fertility treatment: A qualitative study. Perspect Psychiatr Care. 2021;57(2):463-72.
18. Boivin J, Scanlan LC, Walker SM. Why are infertile patients not using psychosocial counselling? Hum Reprod. 1999;14(5):1384-91.
19. Peters E, Vastfjall D, Slovic P, Mertz CK, Mazzocco K, Dickert S. Numeracy and decision making. Psychol Sci. 2006;17(5):407-13.

For peer review only

**Figure legends**

Figure 1- Pictoral representation of the novel intrauterine device and on how it will sit within the uterus. The sensor is batteryless and is powered using a belt and a mobile phone. A belt worn around the waist delivers energy to the sensor and collects data when required. A smartphone controls the system and collects the data.

For peer review only

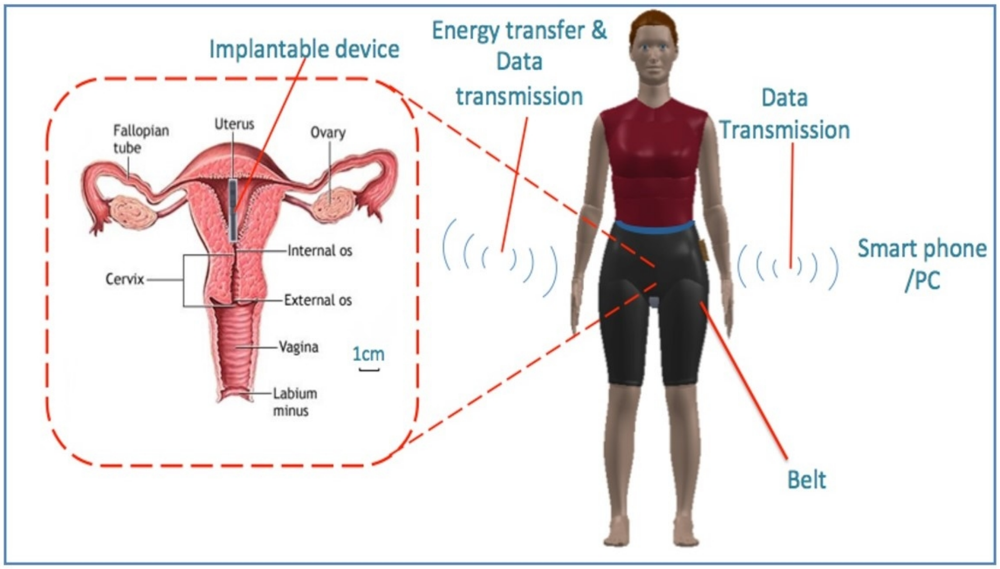


Figure 1- Pictoral representation of the novel intrauterine device and on how it will sit within the uterus. The sensor is batteryless and is powered using a belt and a mobile phone. A belt worn around the waist delivers energy to the sensor and collects data when required. A smartphone controls the system and collects the data.

202x120mm (144 x 144 DPI)

University of Southampton · VivoPlex · NHS University Hospital Southampton NHS Foundation Trust · NHS National Institute for Health Research

# Investigations for women with reproductive problems.

# A questionnaire study.

# What are patients' preferences for a novel device able to monitor the womb environment?

**Introduction**

Doctors and researchers at the University of Southampton in collaboration with VivoPlex Medical Ltd are developing and carrying out research into a novel monitoring device that may be offered to women with fertility issues. Whilst this device has not yet been produced, we are interested in your views about this new sensor device, and what features would be important to you. Participating in this would involve you completing the attached questionnaire. Once this questionnaire is complete and handed to a member of staff, you are not required to do anything further. Responses will be anonymous and confidential. Your views would help us to develop and tailor this novel sensor for women planning a pregnancy in the future. <u>It is important to understand this device is not yet available to you.</u>

We would like you to <u>*imagine*</u> that your doctor has given you the option of using a sensor device in order to find out more about your womb environment. Once the sensor device is inserted into the womb, you would also be asked to wear a garment close to your womb, for a number of days, that holds a small battery pack and information receiver, all supplied to you along with the sensor. The sensor works by measuring three characteristics of your womb environment continuously, and sending that information back to the receiver. Your doctor would discuss the information with you which may allow them to guide you in making decisions about your next treatment steps towards a successful pregnancy.


For peer review only - http://bmjopen.bmj.com/site/about/guidelines.xhtml

University of Southampton VivoPlex NHS University Hospital Southampton NHS Foundation Trust NHS National Institute for Health Research

## Patient information about the study

### What is the purpose of the study?

This is a questionnaire study to help us understand what women consider the most important features of the device. By completing the following questionnaire you could help us to develop important features of this novel test.

### Why have I been chosen and do I need to take part?

As you are attending the outpatient clinic and hoping to have a baby in the future, we would be interested in your opinions about this novel device. We are asking if you would be prepared to complete this questionnaire. You can decide whether or not to take part, and your decision will have no impact on your care.

### What will happen to me if I decide to take part?

Completing the attached questionnaire will take around 10 minutes, and once completed you should return it to the 'questionnaire collection box' at reception. There will be no follow up after the questionnaire has been handed in, and your answers will be confidential and anonymous, and accessed only by members of the research team. The questionnaire asks questions around sensitive topics including fertility and conception and if you do not feel comfortable answering them, you can withdraw from the study at any time without your clinical care being affected.

### What will happen to the results of the research?

The findings will be presented at scientific meetings, nationally and internationally, published in medical journals, and possibly in the local and national press. You will not be identified in these reports or publications. The University policy for data protection (https://www.southampton.ac.uk/about/governance/policies/privacy-policy.page) will be followed.

### Who is organising and funding the research?

This study is funded by the National Institute for Health Research (NIHR) Invention for Innovation stream which supports clinical development of medical devices in areas of existing or emerging patient need. The study is being led by Professor Ying Cheong from the University of Southampton. This study has been reviewed and approved by X Ethics Committee (Ethics No.: to be confirmed).

### Contact for further information.

For further information please contact Dr Bonnie Ng at the University of Southampton, email: bonnie.ng@soton.ac.uk. Or ask to speak to a member of the reproductive health research team 023 8120 6856


For peer review only - http://bmjopen.bmj.com/site/about/guidelines.xhtml

University of Southampton | VivoPlex | NHS University Hospital Southampton NHS Foundation Trust | NHS National Institute for Health Research

**Information on your data**

The University of Southampton is the sponsor or this study based in the United Kingdom, Southampton. We will be using information from you in order to undertake this study and will act as the data controller for this study. This means that we are responsible for looking after your information and using it properly. The University of Southampton will keep identifiable information about you for 10 years after the study has finished.

Your rights to access, change or move your information are limited, as we need to manage your information in specific ways in order for the research to be reliable and accurate. If you withdraw from the study, we will keep the information about you that we have already obtained. To safeguard your rights we will use the minimum personally identifiable information possible.

You can find more about how we use your information at:

http://www.calendar.soton.ac.uk/sectionIV/research-data-management.html

The University of Southampton will collect information from you for this research study in accordance with our instructions.

The University Hospitals Southampton NHS Foundation Trust will keep your name, and contact details confidential and will not pass this information to the University of Southampton. The University Hospitals Southampton NHS Foundation Trust will use this information as needed, to contact you about the research study, and make sure that relevant information about the study is recorded for your care, and to oversee the quality of the study. Certain individuals from the University of Southampton and regulatory organisations may look at your medical and research records to check the accuracy of the research study. The University of Southampton will only receive information without any identifying information The people who analyse the information will not be able to identify you and will not be able to find your name or contact details.

The University Hospitals Southampton NHS Foundation Trust will keep identifiable information about you from this study for 10 years after the study has finished.

**Please pull off the front 2 pages and keep for future reference**


For peer review only - http://bmjopen.bmj.com/site/about/guidelines.xhtml

For peer review only - http://bmjopen.bmj.com/site/about/guidelines.xhtml
For peer review only

University of Southampton · VivoPlex · NHS University Hospital Southampton NHS Foundation Trust · NHS National Institute for Health Research

**CONSENTING TO THE QUESTIONNAIRE.**

If you have any further questions please ask a member of staff to contact the research team on 02381206856.

By ticking this box ☐ you confirm that:

- You have read the information at the start of this questionnaire.
- You understand who to contact with any questions.
- You understand participation in completely voluntary.
- You are over 18 and under 50 years old.
- You are attending an outpatients appointment at the Princess Anne Hospital.

**Study ID** ☐

**When you have completed this questionnaire, please return to the 'Questionnaire collection box' at reception.**

**If you have partially completed the questionnaire or prefer not to take part, you can also return your questionnaire in the same box.**

**Thank you for your time**


For peer review only - http://bmjopen.bmj.com/site/about/guidelines.xhtml

## EXPLANATION ABOUT THE QUESTIONNAIRE.

In this questionnaire you will be presented with several **hypothetical** scenarios in which two sensors with different characteristics will be described. In each situation, we would like you to choose which sensor you prefer out of two options: Device **A** or Device **B**.

Each sensor will be described to you in terms of four features:

1. **Length of time using the device.** The number of days the device would remain inside your womb. Longer time in your womb could give more information to the doctor. There will be three choices, ***7 days, 14 days, or 28 days***

2. **Information obtained from the device and its use in guiding treatment.** There are three choices, the information obtained will guide treatment in a ***limited*** number of cases, ***majority*** of cases or ***all*** cases.

3. **Risk of complications.** There may either be short- or long-term complications from using the device. The long-term effects of using the device are currently unknown, and further clinical trials will be conducted to explore this. The short-term complications could be an infection needing antibiotic treatment, pain or discomfort needing pain relief or expulsion (device falling out of the womb). There will be two choices, 1 in 100 women (***1%, lower risk***), or 10 in 100 women (***10%, higher risk***).

4. **Discreteness of the information receiver.** Once the sensor is inserted into the womb, you will be asked to wear a garment which holds the information receiver. This receiver is required for the data on the womb environment to be obtained and interpreted by a clinician. There are three options regarding the discreteness if the information receiver that you will be asked to wear- ***completely discrete*** (i.e. even when tight clothing is worn the receiver will not be noticeable to others), ***moderately discrete*** (i.e. the receiver will be noticeable to others when tight clothing is worn), and ***indiscrete*** (i.e. the receiver will be noticeable to others irrespective of the type of clothing worn).


For peer review only - http://bmjopen.bmj.com/site/about/guidelines.xhtml

## HOW TO COMPLETE THIS QUESTIONNAIRE.

**<u>There are no right or wrong answers</u>** - we are just interested in your views. Please choose the option that in your opinion is the better device, the one that best matched your priorities.

## EXAMPLE QUESTION

We will now take you through an example question. You will not need to answer the question in this section.

Consider the devices described by the following two sets of features (Device **A** and Device **B**). Please indicate which choice you would prefer by circling the column.

| | **Device A** | **Device B** |
|---|---|---|
| **Length of time using the device** | 7 Days | 28 Days |
| **Information obtained from the device and its use in guiding treatment** | The information obtained will guide treatment in <u>all</u> cases | The information obtained will guide treatment in <u>all</u> cases |
| **Risk of complications** | 10% | 1% |
| **Discreteness of the information receiver** | Moderately discrete | Completely discrete |

In this example, Device A has been selected. The person made the choice based on the features of Device A and Device B, and decided her preferred choice.

## CHOICE SETS


For peer review only - http://bmjopen.bmj.com/site/about/guidelines.xhtml

Please answer **ALL** 9 choice sets and indicate your choice by drawing a circle around your preferred device.

| Choice Set 1 | | |
|---|---|---|
| | **Device A** | **Device B** |
| **Length of using the device** | 7 Days | 28 Days |
| **Information obtained from the device and its use in guiding treatment** | The information obtained will guide treatment in a limited number of cases | The information obtained will guide treatment in a limited number of cases |
| **Risk of complications** | 1% | 10% |
| **Discreteness of the information receiver** | Completely discrete | Indiscrete |

| Choice Set 2 | | |
|---|---|---|
| | **Device A** | **Device B** |
| **Length of using the device** | 28 Days | 7 Days |
| **Information obtained from the device and its use in guiding treatment** | The information obtained will guide treatment in the majority of cases | The information obtained will guide treatment in the majority of cases |
| **Risk of complications** | 10% | 1% |
| **Discreteness of the information receiver** | Completely discrete | Moderately discrete |

| Choice Set 3 | | |
|---|---|---|
| | **Device A** | **Device B** |
| **Length of using the device** | 7 Days | 14 Days |
| **Information obtained from the device and its use in guiding treatment** | The information obtained will guide treatment in a limited number of cases | The information obtained will guide treatment in a limited number of cases |
| **Risk of complications** | 1% | 10% |
| **Discreteness of the information receiver** | Indiscrete | Completely discrete |

| Choice Set 4 | | |
|---|---|---|
| | **Device A** | **Device B** |


For peer review only - http://bmjopen.bmj.com/site/about/guidelines.xhtml

UNIVERSITY OF Southampton
VivoPlex
NHS University Hospital Southampton NHS Foundation Trust
NHS National Institute for Health Research

| | | |
|---|---|---|
| **Length of using the device** | 28 Days | 14 Days |
| **Information obtained from the device and its use in guiding treatment** | The information obtained will guide treatment in a limited number of cases | The information obtained will guide treatment in all cases |
| **Risk of complications** | 10% | 10% |
| **Discreteness of the information receiver** | Moderately discrete | Completely discrete |

| Choice Set 5 | | |
|---|---|---|
| | **Device A** | **Device B** |
| **Length of using the device** | 14 Days | 28 Days |
| **Information obtained from the device and its use in guiding treatment** | The information obtained will guide treatment in all cases | The information obtained will guide treatment in all cases |
| **Risk of complications** | 1% | 10% |
| **Discreteness of the information receiver** | Moderately discrete | Completely discrete |

| Choice Set 6 | | |
|---|---|---|
| | **Device A** | **Device B** |
| **Length of using the device** | 7 Days | 28 Days |
| **Information obtained from the device and its use in guiding treatment** | The information obtained will guide treatment in all cases | The information obtained will guide treatment in all cases |
| **Risk of complications** | 10% | 1% |
| **Discreteness of the information receiver** | Moderately discrete | Indiscrete |


For peer review only - http://bmjopen.bmj.com/site/about/guidelines.xhtml

UNIVERSITY OF Southampton VivoPlex

NHS University Hospital Southampton NHS Foundation Trust
NHS National Institute for Health Research

| Choice Set 7 | | |
|---|---|---|
| | **Device A** | **Device B** |
| **Length of using the device** | 14 Days | 28 Days |
| **Information obtained from the device and its use in guiding treatment** | The information obtained will guide treatment in the majority of cases | The information obtained will guide treatment in the majority of cases |
| **Risk of complications** | 10% | 1% |
| **Discreteness of the information receiver** | Indiscrete | Moderately discrete |

| Choice Set 8 | | |
|---|---|---|
| | **Device A** | **Device B** |
| **Length of using the device** | 14 Days | 7 Days |
| **Information obtained from the device and its use in guiding treatment** | The information obtained will guide treatment in a limited number of cases | The information obtained will guide treatment in a limited number of cases |
| **Risk of complications** | 1% | 10% |
| **Discreteness of the information receiver** | Moderately discrete | Completely discrete |

| Choice Set 9 | | |
|---|---|---|
| | **Device A** | **Device B** |
| **Length of using the device** | 14 Days | 14 Days |
| **Information obtained from the device and its use in guiding treatment** | The information obtained will guide treatment in a limited number of cases | The information obtained will guide treatment in all cases |
| **Risk of complications** | 1% | 1% |
| **Discreteness of the information receiver** | Indiscrete | Completely discrete |


For peer review only - http://bmjopen.bmj.com/site/about/guidelines.xhtml

University of Southampton | VivoPlex | NHS University Hospital Southampton NHS Foundation Trust | NHS National Institute for Health Research

## Section 4: ABOUT YOURSELF

**Age** ☐ **First 4 characters of your postcode** ☐☐☐☐

**Relationship status**

Single ☐
In a relationship and living apart ☐
In a relationship and living together ☐
Engaged ☐
Married ☐
Civil partnership ☐
Other ☐
Please state________________________

**Education attainment**

Higher education & professional/vocational equivalents ☐
A levels, vocational level 3 and equivalents ☐
GCSE/O level grade A*-C, vocational level 2 and equivalents ☐
Qualifications at level 1 and below ☐
Other qualifications: level unknown (including foreign qualifications) ☐
No qualifications ☐

**What is your ethnic group?**

White ☐
Mixed/multiple ethnic groups ☐
Asian/Asian British ☐
Black/African/Caribbean/Black British ☐
Other Ethnic group ☐
Please state______________________________

**Are you needing treatment for infertility/subfertility?**.

Yes ☐ No ☐

**Are you needing treatment for recurrent miscarriages (3 or more consecutive early miscarriages)?**

Yes ☐ No ☐

**Do you or your partner have children?**

Yes ☐ No ☐


For peer review only - http://bmjopen.bmj.com/site/about/guidelines.xhtml

**Have you had any of the following treatment?**

Hysteroscopy (procedure to look inside the womb) ☐

Diagnostic laparoscopy (keyhole procedure to look inside the abdomen only) ☐

Operative laparoscopy (keyhole procedure to look inside the abdomen AND treat pathology) ☐

NHS funded IVF ☐

Privately funded IVF ☐

**Do you qualify for NHS funded IVF?**

Yes ☐ No ☐ Don't know ☐

For peer review only

## Section 5: FEEDBACK (optional)

**How easy or difficult did you find it to answer these questions?** Please put an 'X' by your response:

| | |
|---|---|
| Very easy | |
| Quite easy | |
| Neither easy nor difficult | |
| Quite difficult | |
| Very difficult | |

**Please write any comments you would like to make about this questionnaire:**

______________________________

______________________________

______________________________

______________________________

______________________________

**Do you have any ideas on how to improve the questionnaire or the product?**

______________________________

______________________________

______________________________

______________________________

______________________________

**Thank you for taking the time to complete this questionnaire. Your answers will help us determine how to better develop a novel sensor device designed for women planning a pregnancy.**



For peer review only - http://bmjopen.bmj.com/site/about/guidelines.xhtml
